# SIRT1 Activation by Lignans Identified via UPLC-qTOF-MS/MS-Based Metabolomic Profiling of *Piper longum* L. Fruit (Long Pepper)

**DOI:** 10.3390/plants14203186

**Published:** 2025-10-16

**Authors:** Van-Hieu Mai, Jun-Li Yang, Thi-Kim-Quy Ha, Jorge-Eduardo Ponce-Zea, Minh Thi Tuyet Le, Ba-Wool Lee, Jin-Pyo An, Won Keun Oh

**Affiliations:** 1 Research Institute of Pharmaceutical Sciences, College of Pharmacy, Seoul National University, Seoul 08826, Republic of Korea; maihieu@snu.ac.kr (V.-H.M.); htkquy@ctu.edu.vn (T.-K.-Q.H.); jepz210689@snu.ac.kr (J.-E.P.-Z.); lethituyetminh19289@gmail.com (M.T.T.L.); ntopjp77@gmail.com (J.-P.A.); 2 CAS Key Laboratory of Chemistry of Northwestern Plant Resources and Key Laboratory for Natural Medicine of Gansu Province, Lanzhou Institute of Chemical Physics, Chinese Academy of Sciences (CAS), Lanzhou 730000, China; yangjl@licp.cas.cn; 3 College of Natural Sciences, Can Tho University, Campus II, Ninh Kieu Ward, Can Tho 90000, Vietnam; 4 College of Pharmacy, Sahmyook University, Seoul 01795, Republic of Korea; paul36@syu.ac.kr

**Keywords:** *Piper longum*, *Piperaceae*, long pepper, neolignans, SIRT1

## Abstract

The fruits of *Piper longum* L. (long pepper), a spice and medicinal plant of the family *Piperaceae*, are widely used in South and Southeast Asian cuisine and traditional medicine, valued for their pungent flavor and aroma. The metabolomic profiling of *P. longum* using UPLC-qTOF-MS/MS provided a comprehensive chemical characterization of this traditional medicinal plant, revealing that lignans and amide alkaloids are the major classes of secondary metabolites. To further investigate its pharmacological potential, the bioactive ethyl acetate fraction was subjected to a SIRT1-targeted chemical investigation. This led to the isolation and structural elucidation of three previously undescribed compounds, a cadinene-type sesquiterpene (**1**) and two *oxo*-neolignan (**2** and **5**), along with four known compounds **3**, **4**, **6**, and **7**. Compounds (**1**–**7**) were evaluated for their ability to modulate p53-dependent transcriptional activity via SIRT1 activation using a luciferase reporter cell-based assay. SIRT1, a NAD^+^-dependent deacetylase, is a crucial regulator of longevity, metabolism, and cellular stress resistance, making it a key target for the treatment of age-related diseases. Compounds **2**–**7** exhibited significant SIRT1 activation, with compound **6** displaying particularly high efficacy, comparable to resveratrol, the most well-known natural SIRT1 activator. This study demonstrates that the discovery of novel chemical scaffolds through bioactivity-guided screening highlights the value of combining advanced metabolomics with pharmacological evaluation. The results support the traditional medicinal use of long pepper and its potential for development into functional foods or pharmaceuticals for healthy aging.

## 1. Introduction

The family *Piperaceae* includes five genera, among which *Piper* and *Peperomia* are considered the most important due to their close association with human life as valuable food resources. In particular, *Piper* species have been used for centuries as culinary spices and traditional medicines in tropical and subtropical regions. Representative examples include black pepper (*Piper nigrum* L.) and long pepper (*Piper longum* L.), both highly valued for their distinctive pungent flavor derived from complex mixtures of alkaloids, amides, and essential oils. Notably, black pepper is one of the most widely traded spices in the world and contains piperine as its principal bioactive compound, which not only imparts its characteristic pungency but also exhibits a range of reported health benefits, including anti-inflammatory, antioxidant, and bioavailability-enhancing effects for other nutrients and drugs. Extensive phytochemical and pharmacological studies have demonstrated that *Piper* species possess a wide variety of biological activities. These include insecticidal and larvicidal effects, as well as antibacterial activity, primarily attributed to alkaloids and amides [1,2,3,4]. Propenylphenols have been reported to exert antifungal and nematocidal activities, further broadening the antimicrobial spectrum of these plants. In addition to antimicrobial effects, other physiological activities, such as inhibition of prostaglandin synthesis, gastroprotective effects, and antioxidant capacity, have also reported, collectively suggesting a potential role in modulating inflammation and promoting gastrointestinal health [5,6,7].

SIRT1 (silent information regulator two ortholog 1), a NAD^+^-dependent deacetylase, plays a pivotal role in regulating metabolic pathways, cellular senescence, and stress responses, all of which contribute to the extension of both health span and overall lifespan [8]. SIRT1 activation also contributes to neuroprotection and cognitive function through multiple interrelated mechanisms [9]. By enhancing the non-amyloidogenic processing of amyloid precursor protein (APP) via increased α-secretase activity, SIRT1 reduces the production and accumulation of amyloid-*β* peptides, which are key pathological factors in Alzheimer’s disease [10]. In parallel, SIRT1 suppresses neuroinflammation by modulating inflammatory signaling pathways, thereby creating a neuroprotective environment less susceptible to chronic damage. These combined effects help preserve synaptic plasticity, maintain neuronal integrity, and support cognitive function. Consequently, sustained SIRT1 activity may lower the risk of developing neurodegenerative diseases such as Alzheimer’s, or slow their progression, ultimately contributing to healthier brain aging [11].

Natural product-derived compounds have been extensively investigated for their ability to activate SIRT1. Among these, polyphenols represent one of the most well-studied classes. Resveratrol, a stilbene found in grapes (*Vitis vinifera*), peanuts, and various berries, is considered the prototypical natural SIRT1 activator [12]. It has been shown to enhance NAD^+^-dependent deacetylase activity, stimulate mitochondrial biogenesis, improve oxidative phosphorylation efficiency, and modulate inflammatory signaling pathways, thereby exerting both metabolic and anti-aging benefits [9]. Structurally related compounds such as pterostilbene (abundant in blueberries and grapes), which possesses higher lipophilicity and improved bioavailability compared to resveratrol, have also demonstrated potent SIRT1-activating effects [13,14]. Similarly, quercetin, a flavonol present in onions (*Allium cepa*), apples (*Malus domestica*), and kale, has been reported to activate SIRT1, enhance antioxidant defenses, and regulate glucose metabolism [15].

Metabolite profiling combined with molecular networking has emerged as a powerful and indispensable approach for the preliminary characterization of metabolites in complex plant extracts. In particular, UPLC-qTOF-MS/MS-based profiling enables high-resolution detection and the semi-quantification of a wide array of small molecules, thereby providing a comprehensive snapshot of the phytochemical landscape of a sample. This approach is especially valuable in natural product discovery, where chemical diversity is high and dereplication is crucial to avoid redundant re-isolation of known compounds [16]. Among the various *Piper* species, *P. longum* has been recognized as an important medicinal herb, traditionally used to treat hyperlipidemia, intestinal disorders, and asthma [17,18]. In the present study, three previously undescribed compounds **1**, **2**, and **5** were identified from the EtOAc fraction of long pepper via bioactivity-guided dereplication assisted by UPLC-qTOF-MS/MS spectrometry. Furthermore, their regulatory activity toward SIRT1 was examined together with that of four known compounds **3**, **4**, **6**, and **7**.

## 2. Results and Discussion

### 2.1. Integration of UPLC-qTOF-MS/MS Profiling and Molecular Networking for Dereplication of Piper longum Fruit Extract

**Figure 1:**
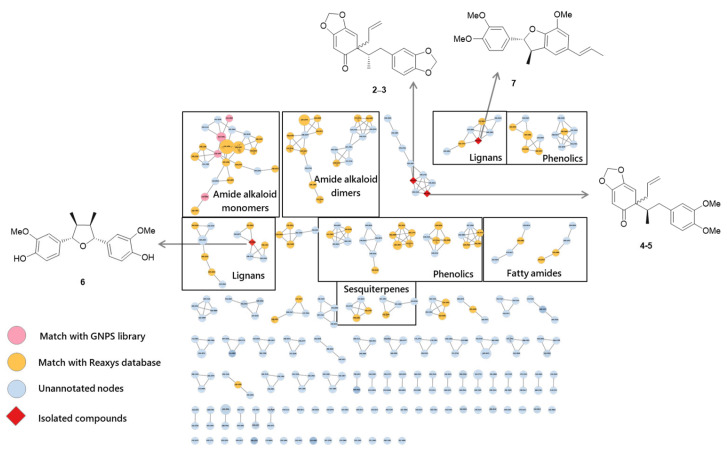
Molecular networking and metabolite profiling of the EtOAc fraction of *P. longum* extract. The network was constructed from UPLC-qTOF-MS/MS data processed with MZmine 4.5. Structurally related metabolites were clustered and classified based on molecular networking results and literature reports. The isolated compounds (**2**–**7**) were manually annotated, providing additional structural insights into the metabolite profile of the EtOAc fraction of *P. longum* extract. Further annotations are provided in Figure S1.

During the search for SIRT1-activating agents, the ethyl acetate (EtOAc) fraction of *Piper longum* fruit (long pepper) exhibited bioactivity, warranting further phytochemical investigation. To elucidate its chemical constituents, the EtOAc-soluble fraction was subjected to UPLC-qTOF-MS/MS analysis under data-dependent acquisition (DDA) mode. The raw spectral data were processed and aligned using MZmine 4.5, after which Feature-Based Molecular Networking (FBMN) was generated. Compound annotation was initially performed through spectral matching against the GNPS (Global Natural Products Social Molecular Networking) database, and the resulting molecular network was visualized using Cytoscape 3.9 (Figure 1). Although the GNPS spectral library represents a valuable community resource, its limited coverage of natural product spectra posed a challenge for exhaustive annotation. Indeed, only a small proportion of nodes within the molecular network could be confidently annotated (highlighted in red in Figure 1), leaving a large portion of the network uncharacterized. To overcome this limitation and improve the annotation rate, we curated a list of previously reported compounds from *P. longum* using the Reaxys online database. This curated reference list was subsequently imported into MZmine 4.5 as a secondary in-house library. Incorporation of this database markedly increased the number of annotated nodes in the FBMN, enabling recognition of distinct chemical clusters corresponding to amide alkaloid monomers and dimers, lignans, phenolics, sesquiterpenes, and fatty amides derivatives (Figure 1 and Figure S1).

Detailed metabolite profiling revealed that amide alkaloid monomers and dimers represented the predominant class of annotated compounds. Specifically, compounds such as piperlonguminine, piperine, piperyline, piperlongumine, dehydropipernonaline, 1-(1-*oxo*-8-hydroxy-2*E*,4*E*-nonadienyl)piperidine, piperlongramide C, piperlongramide E, chabamide, nigramide B, and dipiperamide F were identified. In addition, several fatty amides were detected, including *N*-isobutyl-2,4-octadienamide, 4-*N*-isobutyldecadienamide, and *N*-2′-methylbutyl-2,4-decadienamide. Among these, piperlonguminine was identified as the most abundant amide derivative within the extract, highlighting its dominance in the amide alkaloid profile. Beyond amide alkaloids, a number of phenolic constituents were also annotated, such as methyl-3-(3,4,5-trimethoxyphenyl)acrylate, 3-(3,4,5-trimethoxyphenyl)propanoic acid, piperonal, methyl piperate, and 3,4-methylenedioxycinamaldehyde. Sesquiterpenes were present as well, though only two members, voleneol and aphanamol II, could be conclusively identified. Moreover, the lignans episesamine and asarinin were annotated, but molecular network still contained numerous uncharacterized nodes, suggesting the presence of additional, as yet undescribed metabolites. This finding underlines the necessity for continued in-depth chemical investigation of *P. longum* extracts to fully resolve their chemical diversity.

**Figure 2:**
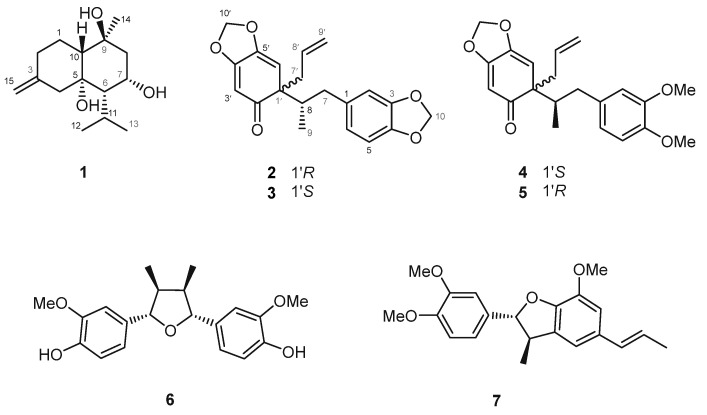
Chemical structures of isolated compounds **1**–**7** from *Piper longum*. Compound **1** was identified as a previously undescribed cadinane-type sesquiterpene bearing hydroxyl groups at C-5 and C-7, with its relative configuration assigned as (5R*, 6S*, 7S*, 9S*, 10R*). Compound **2** was characterized as a new lignan derivative with a unique substitution pattern, elucidated through detailed spectroscopic analyses, including 1D and 2D NMR as well as HRESIMS. Compounds **3**, **4**, **6**, and **7** were identified as known lignans previously reported from natural sources, and their structures were confirmed by comparison of their spectroscopic data with values reported in the literature.

Guided by these metabolomics results, the EtOAc fraction was further subjected to classical chromatographic separation and purification. This effort led to the isolation of seven compounds, comprising three previously undescribed compounds (**1**, **2**, and **5**), and four known lignans (**3**, **4**, **6**, and **7**) (Figure 2). Notably, of these seven isolated compounds, six lignans (**2**–**7**) were also detected and annotated within the assistance of FBMN (Figure 1), thereby validating the reliability of the network-based dereplication workflow and reinforcing the utility of integrating GNPS annotation with curated reference libraries for natural product discovery.

### 2.2. Structure Elucidation of the Previously Undescribed Compounds ***1*** and ***2***

**Figure 3:**
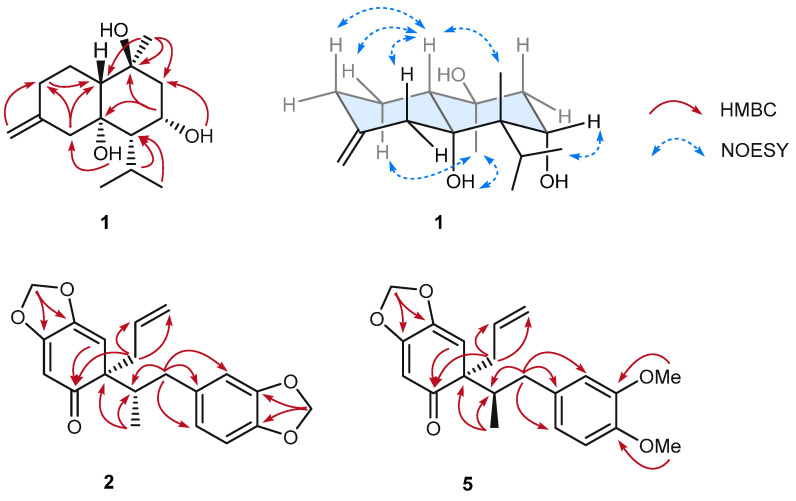
Key HMBC (red arrows) and ROESY (blue dashed arrows) correlations of compound **1**.

Compound **1**, a brownish gum, was assigned the molecular formula C_15_H_26_O_3_, as established by HRESI, which showed a positive molecular ion peak at *m*/*z* 255.1951 [M + H]^+^ (calcd for C_15_H_27_O_3_, 255.1955). The ^13^C NMR spectrum (Table 1) displayed 15 carbon signals, including two olefinic carbons at *δ*_C_ 146.6 and 110.7, three methyl carbons at *δ*_C_ 26.4, 24.3, and 20.5, three oxygenated carbons at *δ*_C_ 79.0, 72.7, and 70.0, four methylene carbons at *δ*_C_ 50.8, 47.5, 35.9, and 23.9, and three methine carbons at *δ*_C_ 56.1, 53.2, and 27.2, consistent with a cadinane-type sesquiterpene skeleton. In the HMBC spectrum (Figure 3), correlations from H-14 (*δ*_H_ 1.30) to C-8 (*δ*_C_ 50.8), C-9 (*δ*_C_ 72.7), and C-10 (*δ*_C_ 56.1), as well as from of H-12 (*δ*_H_ 1.06), H-13 (*δ*_H_ 1.21), and H-11 (*δ*_H_ 2.24) to C-6 (53.2), confirmed that the isopropyl group is attached to C-6 and a methyl group to C-9. Comparison with literature data revealed that ^1^H and ^13^C NMR spectra of compound **1** were similar to those of (+)-isocalamendiol, except for chemical shifts at C-7 (*δ*_C_ 70.0) and C-8 (*δ*_C_ 50.8) (Table 1) [19]. These differences were attributable to the presence of an additional hydroxyl group at C-7. To determine the positions of the hydroxyl groups, the ^1^H NMR spectrum recorded in CD_3_OD was compared with that in DMSO-*d*_6_, revealing two additional peaks assignable to hydroxyl protons. In the HMBC spectrum, one hydroxyl proton showed correlations with C-4 (*δ*_C_ 47.5), C-5 (*δ*_C_ 79.0), and C-6 (*δ*_C_ 53.2), while the other correlated with C-6 and C-7 (*δ*_C_ 70.0), confirming their attachment at C-5 and C-7, respectively. This assignment was further supported by comparison of the ^13^C NMR chemical shifts with previously reported data.

The relative configuration of compound **1** was determined as (5R*, 6S*, 7S*, 9S*, 10R*) through analysis of the ROESY spectrum (Figure 3) in combination with a Chem3D molecular model. The absence of a ROESY correlation between H-10 (*δ*_H_ 1.54) and 5-OH (*δ*_H_ 4.36) (Figure S10) indicated that the decalin is fused in a *trans* configuration. The observed correlation between H-10 and H-6 (*δ*_H_ 1.31) provided additional evidence for the *trans*-fused conformation. Furthermore, the correlation between H-10 and H-11 (*δ*_H_ 2.24) suggested that 5-OH and the isopropyl group share the same orientation. The coupling constant of H-7 (ddd, *J* = 2.9, 2.2 Hz) further indicated that H-7 (*δ*_H_ 4.37) adopts a gauche conformation relative to surrounding protons. Finally, the correlation between 5-OH and H-14 (*δ*_H_ 1.30) confirmed that 5-OH and the Me-14 are positioned on the same molecular face. Based on these data, the relative configuration of compound **1** was assigned as (5R*, 6S*, 7S*, 9S*, 10R*). However, due to the lack of suitable chromophores in the ECD spectrum and the limitations of both Mosher’s method and ECD analysis in this case, the absolute configuration could not be determined.

**Figure 4:**
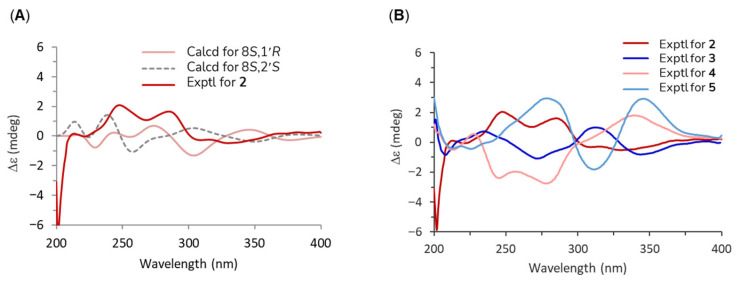
Experimental and calculated ECD spectra of compounds **2**–**5**. (**A**) Theoretical ECD curves of the possible stereoisomers (8*S*,1′*R* and 8*S*,1′*S*) were generated using time-dependent density functional theory (TD-DFT) at the B3LYP/6-31G(d,p) level with the IEFPCM solvent model in MeOH. The calculated spectra were obtained after Boltzmann averaging of conformers based on their relative Gibbs free energies (ΔG). (**B**) The experimental ECD spectra of compounds **2**–**5** were recorded in methanol. Compounds **2** and **4** exhibited opposite Cotton effects. Inversions in the Cotton effect were also observed for compounds **3** and **5**.

Compound **2**, also a brownish gum, had the molecular formula C_20_H_20_O_5_, as determined by HRESIMS, which showed a positive ion at *m*/*z* 341.1385 [M + H]^+^ (calcd for C_20_H_21_O_5_, 341.1384). The ^1^H NMR spectrum exhibited signals for three aromatic protons at *δ*_H_ 6.71 (d, *J* = 7.9 Hz), 6.66 (d, *J* = 1.4 Hz), and 6.61 (dd, *J* = 7.9, 1.4 Hz); three olefinic protons at *δ*_H_ 5.64 (s), 5.59 (s), and 5.55 (m); an exomethylene at *δ*_H_ 5.02 (dd, *J* = 17.0, 1.6 Hz) and 4.94 (dt, *J* = 10.1, 0.9 Hz), two methylenedioxy groups at *δ*_H_ 5.91 (s) and 5.89 (s); one methyl group at *δ*_H_ 0.62 (d, *J* = 6.1 Hz); two pairs of diastereotopic methylene protons at *δ*_H_ 2.97 (d, *J* = 10.3 Hz), 2.10 (m), 2.64 (dd, *J* = 13.0, 7.4 Hz), and 2.57 (dd, *J* = 13.1, 7.1 Hz); and one methine at *δ*_H_ 2.11 (m). The ^13^C NMR spectrum displayed 20 signals which, together with the HSQC data, indicated the presence of one ketone carbon at *δ*_C_ 206.0, one exomethylene carbon at *δ*_C_ 118.4, and two methylenedioxy carbons at *δ*_C_ 102.1 and 103.7. The NMR data were identical to those previously reported for 2′-*oxo*-8,1′-neolignan in the same solvent, suggesting the same planar structure (Table 1). Notably, comparison of the experimental ECD spectrum of compound **2** with that of its stereoisomer **3** revealed distinct differences, indicating a diastereomeric relationship between the two compounds (Figure 4B). To determine the absolute configuration of compound **2**, two possible diastereomers at C-8 and C-1′ were considered for theoretical ECD calculations. Time-dependent density functional theory (TD-DFT) calculations at the B3LYP/6-31G (d,p) level, using the IEFPCM solvent model in MeOH, were performed. The simulated ECD curves of the 8*S*,1′*R* and 8*S*,1′*S* isomers were generated after Boltzmann averaging of conformers based on free Gibbs energy (ΔG) (Tables S2 and S3). The calculated ECD spectrum of the 8*S*,1′*R* isomer matched the experimental spectrum of compound **2** (Figure 4A). Based on these results, compound **2** was identified as a new isomer of Δ^8′^-l′,2′-dihydro-3,4,3′,4′-*bis*-methylenedioxy-2′-*oxo*-8*S*,1′*R*-neolignan.

Compound **5** was obtained as a brownish gum. Its molecular formula was established as C_21_H_24_O_5_ by HRESIMS, which showed a sodium adduct ion at *m*/*z* 379.1510 [M + Na]^+^ (calcd for C_21_H_24_O_5_Na, 379.1516). Compared with compound **2**, the ^1^H NMR spectrum of compound **5** showed only one methylenedioxy signal at *δ*_H_ 5.92 (s), while two additional methoxy signals appeared at *δ*_H_ 3.79 (s) and 3.78 (s), suggesting that one methylenedioxy group was replaced by two methoxy groups. The positions of these two methoxy groups were assigned to C-3 and C-4, as evidenced by correlations from 3-OMe to C-3 and 4-OMe to C-4 (Figure 3). Comparison with literature data indicated that the planar structure of compound **5** corresponded to cymosalignan B (**4**) [20], except for notable deviations in the chemical shifts at the stereogenic centers at C-8 and C-1′, suggesting a stereoisomeric relationship. Since the molecular scaffolds of compounds **2**/**3** and **4**/**5** are identical, differing only in the substituents at C-3 and C-4, these variations were not expected to significantly affect the Cotton effect. Accordingly, compounds **2**–**5** were predicted to exhibit comparable ECD profiles if their configurations were the same. The absolute configuration of cymosalignan B (**4**) was first established in this study, as it had not been reported previously. The experimental ECD spectrum of compound **4** (Figure 4B) was nearly opposite in sign to that of compound **2**, indicating an inverted stereochemistry, thus, compound **4** was assigned as 8*R*,1′*S*-**4**. Similarly, the experimental ECD spectrum of **5** displayed an inverted Cotton effect compared with compound **3**, supporting the assignment of an absolute configuration of 8*R*,1′*R*-**5**. Based on these findings, compound **5** was determined to be a new neolignan and was designated as isocymosalignan B.

The structures of the remaining four known compounds were elucidated by comparing their spectroscopic data with those reported in the literature, and were identified as Δ^8′^-l′,2′-dihydro-3,4,3′,4′-*bis*-methylenedioxy-2′-oxo-8*S*,1′*S*-neolignan (**3**), cymosalignan B (**4**) [20], nectandrin B (**6**) [21], and acuminatin (**7**) [22].

### 2.3. SIRT1-Stimulatory Effects of Neolignans Isolated from Piper longum Fruit

**Figure 5:**
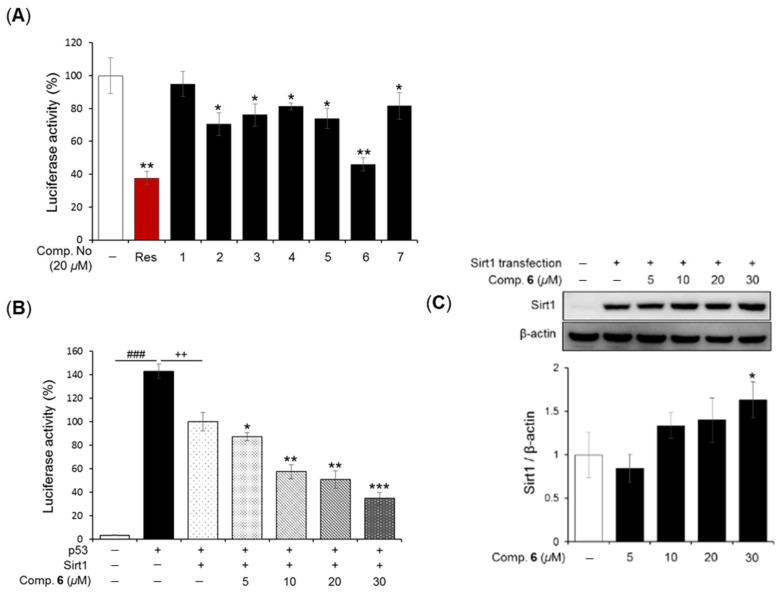
Effects of the isolated compounds from *P. longum* on SIRT1 deacetylation activity and SIRT1 expression in HEK293 cells. (**A**) HEK293 cells were transfected with RSV-*β*-gal, PG13-luc, myc-p53, and flag-SIRT1 plasmids for 24 h. The cells were then treated with seven compounds (20 μM) and resveratrol as a positive control. After 12 h of incubation, cell lysates were collected, and p53 transcriptional activity was measured using a luciferase reported assay. Data are expressed as the mean ± SD (*n* = 3), * *p* < 0.05, ** *p* < 0.01, and *** *p* < 0.001 compared with the negative control. (**B**) The effect of compound **6** on SIRT1 deacetylation activity was evaluated at different concentrations (5 to 30 µM). Data are presented as the mean ± SD (*n* = 3); ^###^ *p* < 0.001 compared with the vehicle group; ++ *p* < 0.05 compared with the p53-positive control; * *p* < 0.05, ** *p* < 0.01, and *** *p* < 0.001 compared with the SIRT1/p53-positive control. (**C**) HEK293 cells were transfected with the Flag-SIRT1 plasmid for 12 h, followed by incubated with compound **6** at various concentrations for 24 h. SIRT1 protein expression was measured by Western blotting. Data are expressed as the mean ± SD (*n* = 2). * *p* < 0.05 compared with the SIRT1-positive control.

In our investigation to identify SIRT1-activating compounds (STACs) from *P. longum*, seven isolated compounds (**1**–**7**) were screened for their ability to modulate p53-dependent transcriptional activity via SIRT1 activation using a luciferase reporter cell-based assay. Mammalian p53 and SIRT1 expression vectors were transfected into HEK293 cells along with a reporter construct containing p53 binding sites upstream of the luciferase gene. After 24 h of transfection, the tested compounds were applied as a concentration of 20 µM, with resveratrol used as a positive control [23,24]. The results demonstrated that compounds **2**–**7** decreased luciferase activity compared with vehicle treatment (Figure 5A). Among them, compound **6** exhibited the strongest activity, showing a trend similar to that of resveratrol. Based on the screening results, compound **6** was further examined for its detailed SIRT1-activating effects. As shown in Figure 5B, compound **6** significantly reduced p53-mediacted transcriptional activation in a concentration-dependent manner (5 to 30 µM).

Given that SIRT1 is an important therapeutic target for age-related diseases [25], we also investigated whether compound **6** could enhance SIRT1 protein expression in HEK293 cells. Cells were treated with compound **6** (5 to 30 µM) for 24 h after transfection with a flag-SIRT1 plasmid. Western blot analysis revealed that transfected cells displayed markedly higher SIRT1 protein levels compared with non-transfected cells (5C). Interestingly, treatment with compound **6** at 30 µM further enhanced SIRT1 protein expression up to 1.63 ± 0.21-fold relative to transfected cells alone (1.00 ± 0.25). Collectively, these results suggest that neolignans from *P. longum* have potential as natural SIRT1 activators through stimulation of SIRT1 deacetylation. Although the chemical constituents and diverse pharmacological activities of *P. longum* have been previously reported [26], this study demonstrates, for the first time, that *P. longum* can be considered a promising natural source of SIRT1 activators for the therapeutic management of age-related diseases.

### 2.4. Molecular Docking Simulation of Compound ***6***

**Figure 6:**
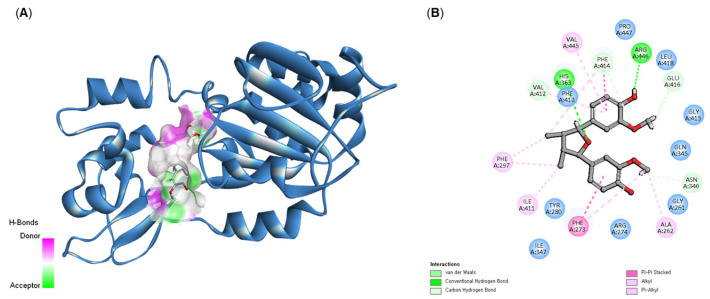
Interaction between compound **6** and the binding pockets of 4I5I. (**A**) Docking poses of compound **6** within the binding pocket. (**B**) Key interactions between compound 6 and the SIRT1 protein.

To investigate the direct stimulatory mechanism of compound **6**, molecular docking studies were performed with the crystal structure of the human SIRT1 protein (PDB 4I5I). Docking calculations were conducted using the CDOCKER module in Discovery Studio to estimate the binding energy of the ligand-protein complex. A lower CDOCKER energy indicates stronger binding affinity. Compound **6** exhibited a CDOCKER energy of −11.06 kcal/mol, whereas resveratrol, the positive control, showed a stronger binding energy of −20.35 kcal/mol. These results were consistent with the experimental findings, suggesting that compound **6** is slightly less potent than resveratrol, the difference is not substantial. Binding affinity within well-defined protein pockets is primarily governed by noncovalent interactions, with hydrogen bonding and electrostatic interactions often contributing most significantly [27,28]. Detailed analysis of the 4I5I protein–ligand docking model revealed two hydrogen bonds: one between the 4-OH group of compound **6** and Arg446, and another between the 7-O group and His363, which help anchor the ligand in the active site. In addition, hydrophobic and van der Waals interactions further stabilized the complex [28]. Notably, Phe273 and Phe414 formed π–π stacking interactions with the aromatic rings of compound 6 (Figure 6B). Moreover, ring A engaged in CH–π interactions with Val414 and Val445. Collectively, these interactions suggest that compound 6 fits stably within the hydrophobic binding pocket of the SIRT1 catalytic domain, supporting its role as a strong binder to this protein target.

## 3. Materials and Methods

### 3.1. General Experimental Procedures

Analytical-grade solvents and HPLC-grade solvents were used for extraction, isolation, and HPLC-MS analysis, respectively. Column chromatography (CC) was performed using silica gel (63−200 μm particle size) purchased from ZEOCHEM (Rüti, Switzerland) and RP-C18 (75 μm particle size) obtained from Nacalai (Kyoto, Japan). Thin-layer chromatography (TLC) was conducted on RP-18 F254 or silica gel 60 F254 plates (Merck, Darmstadt, Germany). HPLC was carried out on a Gilson system equipped with a UV detector and a YMC C18 column (10 × 250 mm, 10 μm particle size; RS Tech, Gimpo, Republic of Korea). Simulated ECD spectra were calculated using Gaussian 16 package. Optical rotations were measured on a JASCO P-2000 polarimeter (JASCO, Hachioji, Japan). NMR spectra of compounds **1**–**7** were recorded in deuterated solvents using a JNM-ECA-600 (JEOL, 600 MHz) spectrometer (JEOL, Tokyo, Japan) and a Gemini 2000 (Varian, Palo Alto, CA, USA, 400 MHz) spectrometer at the College of Pharmacy, Seoul National University, Seoul, Korea. Experimental CD spectra were measured on a Chirascan plus instrument (Applied Photophysics Ltd., Surrey, UK).

### 3.2. Plant Material

The dried fruits of *Piper longum* L. were purchased from a local market in Seoul, Republic of Korea, and authenticated by Prof. Dr. Won-Keun Oh (College of Pharmacy, SNU). A voucher specimen (SNU-1507) was deposited in the Herbarium of the Medicinal Plant Garden, College of Pharmacy, SNU.

### 3.3. Extraction and Isolation

The dried fruits of *P. longum* (3 kg) were extracted with MeOH at room temperature for 48 h (8 L × 3). The combined extract was concentrated under reduced pressure to yield a dried residue, which was suspended in H_2_O and partitioned successively with *n*-hexane and EtOAc. The EtOAc-soluble fraction (31 g) was subject to silica gel open-column chromatography (OCC) eluted with an n-hexane/acetone gradient (20/1 → 0/1) to afford five fractions (E1–E5). Fraction E2 (3.1 g) was further purified on a C18 column using a MeOH/H_2_O gradient (3/1 → 0/1) to yield four subfractions (E2.1–E2.5). Subfraction E2.1 (350 mg) was purified by preparative HPLC (Gilson, C18, 10 × 250 mm, Optima Pak, RS Tech) using 70% MeOH/H_2_O containing 0.1% formic acid (3 mL/min, 30 min) to afford compounds **2** (18 mg) and **3** (20 mg). Subfraction E2.3 (180 mg) was purified by reversed phase HPLC (C18, 10 × 250 mm, Optima Pak, RS Tech) using 65% MeOH/H_2_O containing 0.1% formic acid (3 mL/min, 25 min) to yield compound **1** (2.2 mg). Fraction E4 (4.2 g) was separated on a silica gel column eluted with hexane/EtOAc (10/1 → 1/1) to give four subfractions (E4.1–E4.4). Subfraction E4.3 afforded compound **6** (120 mg), while subfraction E4.1 (205 mg) was purified by HPLC (C18, 10 × 250 mm, Optima Pak, RS Tech) using 85% MeOH/H_2_O containing 0.1% formic acid (3 mL/min, 25 min) to yield compound **7**. Subfraction E4.2 (321 mg) was further purified by HPLC (C18, 10 × 250 mm, Optima Pak, RS Tech) using 72% MeOH/H_2_O containing 0.1% formic acid (3 mL/min, 40 min) to afford compounds **4** (52 mg) and **5** (48 mg).

#### 3.3.1. 7-Hydroxyisocalmendiol (1)

Brownish gum; $\alpha_{D}^{20}$ = −0.9 (c 0.1, MeOH); UV (MeOH) *λ*_max_ (log ε) 210 (3.2); ^1^H and ^13^C NMR data, see Table 1; HRESIMS *m*/*z* 255.1951 [M + H]^+^ (calcd for C_15_H_27_O_3_, 255.1955).

#### 3.3.2. Δ^8′^-l′,2′-Dihydro-3,4,3′,4′-*bis*-methylenedioxy-2′-*oxo*-8*R*,1*S*′-neolignan (2)

Brownish gum; $\alpha_{D}^{20}$ = +14.2 (c 0.5, MeOH); UV(MeOH) *λ*_max_ (log ε) 245 (2.8), 290 (2.6); ^1^H and ^13^C NMR data, see Table 1; HRESIMS *m*/*z* 341.1385 [M + H]^+^ (calcd for C_20_H_21_O_5_, 341.1384).

#### 3.3.3. Isocymosalignan B (3)

Brownish gum; $\alpha_{D}^{20}$ = +23.2 (c 0.5, MeOH); UV(MeOH) *λ*_max_ (log ε) 247 (2.7), 293 (2.6); ^1^H and ^13^C NMR data, see Table 1; HRESIMS *m*/*z* 379.1510 [M + Na]^+^ (calcd for C_21_H_24_O_5_, 379.1516).

### 3.4. ECD Calculations

The structure was constructed using ChemDraw 21 and subjected to conformational searches with the MMFF94 force field in Gaussian 16, applying an energy cut-off of 2.0 kcal/mol to select relevant conformers. Only conformers with populations greater than 1% were retained for further refinement. These conformers were subsequently optimized, and frequency calculations were performed at the B3LYP/6-31G(d) level of theory using Gaussian 16. Optimized conformers with a Boltzmann population above 1% were then used for electronic circular dichroism (ECD) spectral calculations. ECD spectra were calculated using time-dependent density functional theory (TDDFT) at the B3LYP/6-31G(d,p) level with the polarizable continuum model (PCM) in methanol, considering 40 excitation states. Theorical ECD spectra were generated with SpecDis version 1.70.1 [29]. Final spectra were obtained by Boltzmann-weighted averaging based on Gibbs free energy, using a Gaussian bandwidth (σ) of 0.3 eV and a UV correction of 0–10 nm.

### 3.5. SIRT 1 Deacetylation Assay with a Luciferase Reporter Cell-Based Assay

The luciferase reporter cell-based assay was performed according to a previously reported protocol with slightly modifications [23]. HEK293 cells were maintained in Dulbecco’s Modified Eagle’s Medium (DMEM; Hyclone, Logan, UT, USA) supplemented with 10% fetal bovine serum (FBS; Hyclone), 100 U/mL penicillin, and 100 μg/mL streptomycin (Gibco, Grand Island, NY, USA) at 37 °C in a humidified atmosphere containing 5% CO_2_. The cells were seeded onto 48-well plates and incubated for 12 h. Plasmids [0.1 µg PG13-luc plasmid (wt p53 binding sites), 0.05 µg myc-tagged p53 (myc-p53), 0.1 µg Flag-tagged SIRT1 (Flag-SIRT1), and 0.1 µg RSV-*β*-galactosidase] were transfected into each well using PEI transfection reagent (Polyscience, Inc., Warrington, PA, USA). After 24 h of transfection, the cells were treated with the tested compounds and further incubated for 12 h at 37 °C and 5% CO_2_. Cell lysates were then collected using passive lysis buffer (Promega, Madison, WI, USA), and luciferase activity was measured using a Dual-Luciferase Reporter Assay Kit (Promega). The activities of the target reporters (p53, myc-p53, and Flag SIRT1) were normalized to constitutive *β*-galactosidase expression. The final data was evaluated by dividing luciferase activity by Renilla luciferase activity.

### 3.6. Western Blot Analysis

HEK293 cells were seeded onto 6-well plates and incubated for 12 h. Cells were then transfected with the Flag-SIRT1 plasmid using PEI transfection reagent for 12 h. The medium was replaced with fresh DMEM containing 5% FBS, 100 U/mL penicillin, and 100 μg/mL streptomycin, and cells were treated with the test compound at various concentrations. After 24 h of incubation, cell lysates were collected using lysis buffer (0.5% NP-40, 50 mM NaF, 1 mM EDTA, 120 mM NaCl, and 50 mM Tris-HCl, pH 7.6) and centrifuged at 12,000 rpm for 20 min. Protein concentrations were determined using a protein assay kit (Bio-Rad Laboratories, Hercules, CA, USA). Aliquots of lysates were electrophoresed on 12% SDS-polyacrylamide gels and transferred onto Immobilon-P PVDF membranes (0.45 μm, Millipore, Darmstadt, Germany). Membranes were incubated overnight at 4 °C with primary antibodies against SIRT1 (ab12193, Abcam, Cambridge, UK) or actin (ab6276, Abcam). After washing, membranes were incubated with the corresponding secondary antibodies for 2 h at room temperature. Protein bands were visualized using an enhanced chemiluminescence (ECL) detection kit (Thermo Scientific, Rockford, IL, USA) and quantified using an LAS 4000 luminescent image analyzer (Fuji Film, Tokyo, Japan).

### 3.7. Molecular Docking Simulation

All docking calculations were performed using BIOVA Discovery Studio 2025 software. The crystal structure of the human SIRT1 protein (PDB ID: 4I5I) was obtained from the RCSB Protein Data Bank (https://www.rcsb.org/structure/4I5I, accessed on 26 March 2025). Protein preparation involved the removal of water molecules and co-crystallized ligands to generate the receptor structure for docking. Ligands were energy-minimized using the MMFF94 force field prior to docking. Molecular docking was performed using the CDOCKER module, and the binding affinities of the docked poses were evaluated based on the CDOCKER scores, expressed in kcal/mol.

## 4. Conclusions

The dereplication-guided isolation of the bioactive EtOAc fraction of *P. longum* fruits (long pepper) afforded one new cadinene-type sesquiterpene and two new *oxo*-neolignan, 7-hydroxyisocalmendiol (**1**), Δ^8′^-l′,2′-dihydro-3,4,3′,4′-*bis*-methylenedioxy-2′-*oxo*-8*R*,1*S*′-neolignan (**2**), and isocymosalignan B, together with four known neolignans and lignan, Δ^8′^-l′,2′-dihydro-3,4,3′,4′-*bis*-methylenedioxy-2′-*oxo*-8*S*,1*S*′-neolignan (**3**), cymosalignan B (**4**), acuminatin (**6**), and nectandrin B (**7**). Notably, the absolute configurations of compound **2**–**5** were fully established using experimental and calculated ECD spectra, thereby complementing the incomplete stereochemical information reported in previous studies. Among them, compound **6** significantly stimulated SIRT1 expression in HEK293 cells, in strong agreement with the molecular docking results. These findings suggest that the bioactive lignans isolated from *P. longum* fruits may serve as promising natural modulators of SIRT1 activity, thereby enhancing the pharmacological potential of *P. longum* in therapeutic strategies for age-related disorders.

## Figures and Tables

**Table 1:** ^1^H (600 MHz) and ^13^C NMR (150 MHz) data for compounds **1**, **2**, and **5** (MeOH-*d*_4_).

	1			2			5	
Position	δ_C_, Type	δ_H_ (*J* in Hz)	Position	δ_C_, Type	δ_H_ (*J* in Hz)	Position	δ_C_, Type	δ_H_ (*J* in Hz)
1	23.9, CH_2_	1.91, m 1.60, m	1	135.9, C	-	1	135.0, C	-
2	35.9, CH_2_	2.40, dt (12.7, 4.1) 2.02, m	2	110.3, CH	6.66, d (1.4)	2	114.1, CH	6.65, d (1.9)
3	146.6, C	-	3	147.3, C	-	3	148.9, C	-
4	47.5, CH_2_	2.58, dd (13.5, 1.4) 2.00, dd (13.2, 0.7)	4	149.1, C	-	4	150.4, C	-
5	79.0, C	-	5	108.9, CH	6.71, d (7.9)	5	113.1, CH	6.80, d (8.1)
6	53.2, CH	1.31, dd (4.3, 1.9)	6	123.1, CH	6.61, dd (7.9, 1.4)	6	122.6, CH	6.60, dd (8.1, 1.8)
7	70.0, CH	4.37, ddd (2.9, 2.2)	7	37.8, CH_2_	2.97, d (10.3) 2.10, m	7	37.6, CH_2_	2.46, m 2.04, dd (13.1, 11.1)
8	50.8, CH_2_	2.11, dd (13.8, 3.5) 1.70, dd (13.8, 2.5)	8	46.6, CH	2.11, m	8	46.4, CH	2.31, m
9	72.7, C	-	9	14.5, CH_3_	0.62, d (6.1)	9	14.6, CH_3_	0.86, d (6.9)
10	56.1, CH	1.54, dd (12.5, 2.4)	10	102.1, CH_2_	5.89, s	3-OMe	56.6, CH_3_	3.79, s
11	27.2, CH	2.24, m	-	-	-	4-OMe	56.5, CH_3_	3.78, s
12	24.3, CH_3_	1.06, d (6.8)	1′	58.6, C	-	1′	58.7, C	-
13	20.5, CH_3_	1.21, d (7.0)	2′	206.0, C	-	2′	206.0, C	-
14	26.4, CH_3_	1.30, s	3′	100.6, CH	5.59, s	3′	100.6, CH	5.65, s
15	110.7, CH_2_	4.75, d (1.9) 4.66, d (1.6)	4′	166.9, C	-	4′	166.9, C	-
			5′	146.8, C	-	5′	146.8, C	-
			6′	107.7, CH	5.64, s	6′	107.8, CH	5.69, s
			7′	44.3, CH_2_	2.64, dd (13.0, 7.4) 2.57, dd (13.1, 7.1)	7′	44.4, CH_2_	2.48, m
			8′	134.3, CH	5.55, m	8′	134.3, CH	5.52, m
			9′	118.4, CH_2_	5.02, dd (17.0, 1.6) 4.94, dt (10.1, 0.9)	9′	118.4, CH	4.98, dd (17.0, 2.1) 4.93, dd (10.1, 2.1)
			10′	103.7, CH_2_	5.91, s	10′	103.6, CH_2_	5.92, s

## Data Availability

The data supporting the findings of this study are available from the corresponding author upon reasonable request.

## Supplementary Materials

The following supporting information can be downloaded at: https://www.mdpi.com/article/10.3390/plants14203186/s1, Figure S1: Additional annotations of known compounds in the molecular network of the EtOAc fraction from *P. longum* extract; Figure S2: (+)-HR-ESI-MS of compound **1**; Figures S3–S6: 1D and 2D-NMR spectra of compound **1** in MeOH-*d*_4_; Figures S7–S10: 1D and 2D-NMR spectra of compound **1** in DMSO-*d*_6_; Figure S11: (+)-HR-ESI-MS of compound **2**; Figures S12–S15, 1D and 2D-NMR spectra of compound **2** in MeOH-*d*_4_; Figure S16: (+)-HR-ESI-MS of compound **5**; Figures S17–S19: 1D and 2D-NMR spectra of compound **5** in MeOH-*d*_4_; Figures S20 and S21: Energy-minimized conformers of 8*S*,1′*R*-**2** and 8*S*,1′*S*-**2** at the B3LYP/6-31G(d) in the gas phase; Table S1: Metabolite annotations of *Piper longum* fruit extract; Tables S2–S3. Relative Gibbs free energy (ΔG) and Boltzmann population of 8*S*,1′*R*-**2** and 8*S*,1′*S*-**2**.
