# Supplementary material for: SIRT1 Activation by Lignans Identified via UPLC-qTOF-MS/MS-Based Metabolomic Profiling of Piper longum L. Fruit (Long Pepper)
Source: Plants (Basel). 2025 Oct 16;14(20):3186. doi: 10.3390/plants14203186 (PMC12566738; doi:10.3390/plants14203186)
Supplement: Supplementary file 1 [file plants-14-03186-s001.zip › plants-3903631-supplementary.pdf]

Supplementary Materials

1. Supplementary data **3**

Figure S1. Additional annotations of known metabolites in the molecular networking of the EtOAc layer from *P. longum* extract. 3

. ... 3

Table S1. Metabolite annotations of *Piper longum* fruits extract 4

2. Spectra of all compounds **7**

2.1 Compound 1 in MeOH-d_4 *7*

Figure S2. (+)-HR-ESI-MS of compound **1** 7

Figure S3. ^{1}H NMR spectrum of compound **1** in MeOH-d_4 (600 MHz) 7

Figure S4. ^{13}C NMR spectrum of compound **1** in MeOH-d_4 (150 MHz) 8

Figure S5. HSQC spectrum of compound **1** in MeOH-d_4 (600 MHz) 8

Figure S6. HMBC spectrum of compound **1** in MeOH-d_4 (600 MHz) 9

2.2 Compound 1 in DMSO-d_6 *10*

Figure S7. ^{1}H NMR spectrum of compound **1** in DMSO-d_6 (600 MHz) 10

Figure S8. ^{13}C NMR spectrum of compound **1** in DMSO-d_6 (150 MHz) 10

Figure S9. HMBC spectrum of compound **1** in DMSO-d_6 (600 MHz) 11

Figure S10. ROESY spectrum of compound **1** in DMSO-d_6 (600 MHz) 11

2.3 Compound 2 in MeOH-d_4 *12*

Figure S11. (+)-HR-ESI-MS of compound **2** 12

Figure S12. ^{1}H NMR spectrum of compound **2** in MeOH-d_4 (600 MHz) 12

Figure S13. ^{13}C NMR spectrum of compound **2** in MeOH-d_4 (150 MHz) 13

Figure S14. HSQC spectrum of compound **2** in MeOH-d_4 (600 MHz) 13

Figure S15. HMBC spectrum of compound **2** in MeOH-d_4 (600 MHz) 14

2.4 Compound 5 in MeOH-d_4 *15*

Figure S16. (+)-HR-ESI-MS of compound **5** 15
Figure S17. ^{1}H NMR spectrum of compound **5** in MeOH-d_4 (600 MHz) 15
Figure S18. ^{13}C NMR spectrum of compound **5** in MeOH-d_4 (150 MHz) 16
Figure S19. HMBC spectrum of compound **5** in MeOH-d_4 (600 MHz) 16
3. ECD Calculations **17**
Table S2. Relative Gibbs free energy (ΔG) and Boltzmann population of 8*S*,1′*R*-**2** 17
Figure S20. Energy-minimized conformers of 8*S*,1′*R*-**2** at the B3LYP/6-31G(d) in the gas phase 17
Table S3. Relative Gibbs free energy (ΔG) and Boltzmann population of 8*S*,1′*S*-**2** 18
Figure S21. Energy-minimized conformers of 8*S*,1′*S*-**2** at the B3LYP/6-31G(d) in the gas phase 18

1. Supplementary data

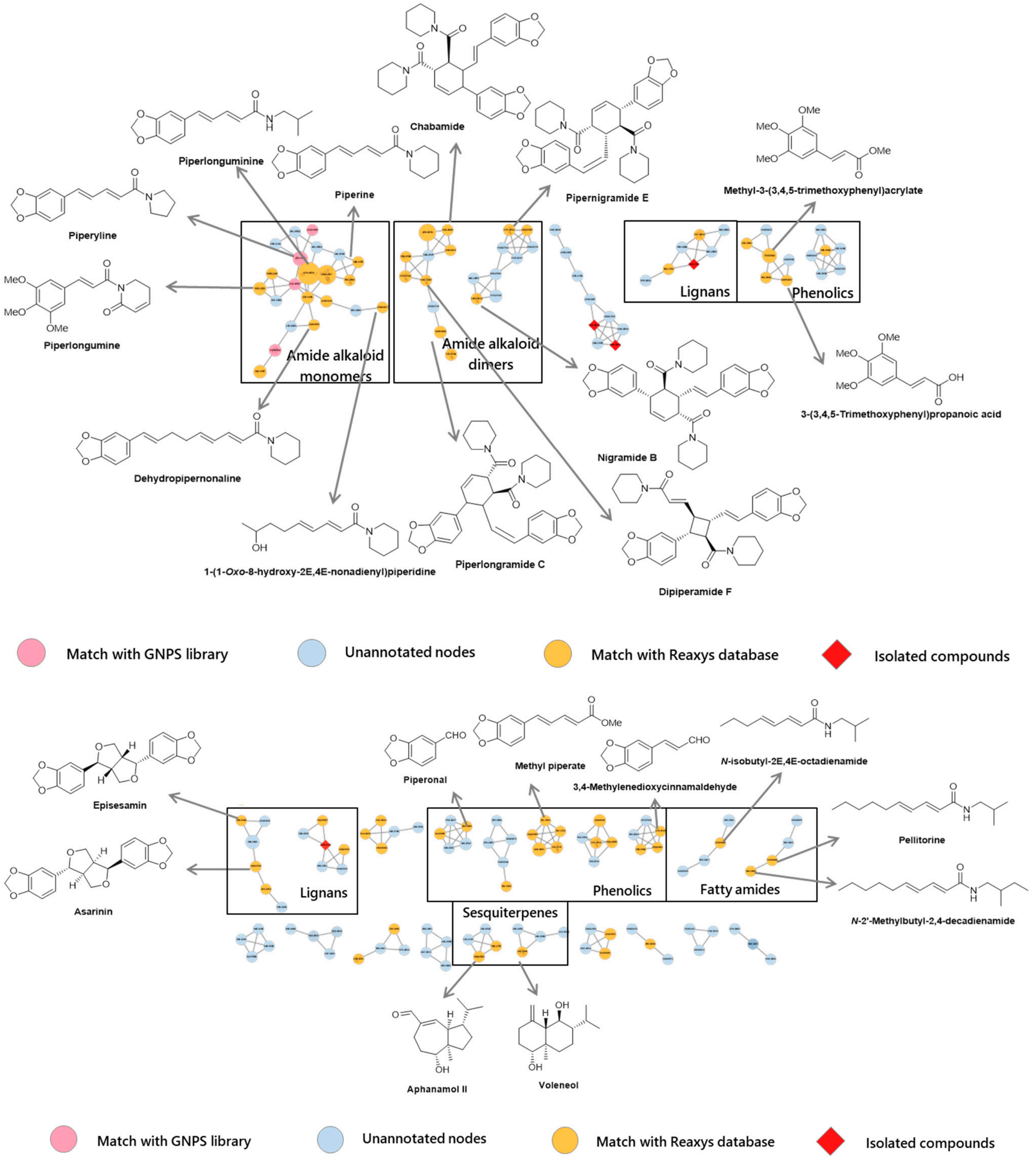

Figure S1. Additional annotations of known metabolites in the molecular networking of the EtOAc layer from *P. longum* extract.

.

Table S1. Metabolite annotations of *Piper longum* fruits extract

Compound name	rt	m/z	Molecular formula	adduct	m/z_diff (ppm)	neutral_mass	SMILE
2-oxoisodauc-5-en-12-al	8.94	252.1939	C15H22O2	$[M+NH_4]^+$	-7.37	234.162	[H][C@@]12C=C(CCC(=O)[C@]1(C)CC[C@H]2C(C)C)C=O
caryophyllenol-II	14.36	238.2161	C15H24O	$[M+NH_4]^+$	-1.89	220.1827	[H][C@]12CC(C)(C)[C@]1([H])C\C=C(C)/[C@@H](O)CCC2=C
longimin A	9.69	264.1961	C16H22O2	$[M+NH_4]^+$	1.06	246.162	C\C(=C/C1=CC(C)(C)OC1=O)[C@H]1CCC(C)=CC1
retrofractamide A	12.57	328.1888	C20H25NO3	$[M+H]^+$	-5.78	327.1834	CC(C)CNC(=O)\C=C\C=C\CC\C=C\C1=CC2=C(OCO2)C=C1
retrofractamide B	14.26	356.2177	C22H29NO3	$[M+H]^+$	-12.14	355.2147	CC(C)CNC(=O)\C=C\C=C\CCCC\C=C\C1=CC2=C(OCO2)C=C1
Guineensine	15.94	406.2330	C24H33NO3	$[M+Na]^+$	-5.46	383.246	CC(C)CNC(=O)\C=C\C=C\CCCCCC\C=C\C1=CC2=C(OCO2)C=C1
brachystamide B	17.48	412.2803	C26H37NO3	$[M+H]^+$	-10.44	411.2773	CC(C)CNC(=O)\C=C\C=C\CCCCCCCC\C=C\C1=CC=C2OCOC2=C1
fagaramide	8.55	248.1293	C14H17NO3	$[M+H]^+$	4.91	247.1208	CC(C)CNC(=O)\C=C\C1=CC=C2OCOC2=C1
Retrofractamide C	13.01	330.2036	C20H27NO3	$[M+H]^+$	-8.41	329.1991	CC(C)CNC(=O)\C=C\CCCC\C=C\C1=CC2=C(OCO2)C=C1
piperchabamide D	14.75	358.2355	C22H31NO3	$[M+H]^+$	-6.01	357.2304	CC(C)CNC(=O)\C=C\CCCCCC\C=C\C1=CC2=C(OCO2)C=C1
Piperlonguminine	7.06	274.1455	C16H19NO3	$[M+H]^+$	6.35	273.1365	CC(C)CNC(=O)C=CC=CC1=CC2=C(OCO2)C=C1
Piperlonguminine (isomer)	9.89	274.1419	C16H19NO3	$[M+H]^+$	-6.92	273.1365	CC(C)CNC(=O)C=CC=CC1=CC2=C(OCO2)C=C1
Piperlonguminine (isomer)	10.09	274.1436	C16H19NO3	$[M+H]^+$	-0.46	273.1365	CC(C)CNC(=O)C=CC=CC1=CC2=C(OCO2)C=C1
dihydropiperlonguminine	6.86	276.1605	C16H21NO3	$[M+H]^+$	3.73	275.1521	CC(C)CNC(=O)C=CCCC1=CC=C2OCOC2=C1
piperonal	15.31	151.0382	C8H6O3	$[M+H]^+$	-5.29	150.0317	O=CC1=CC=C2OCOC2=C1
Methyl-3-(3,4,5-trimethoxyphenyl)acrylate	16.32	253.1062	C13H16O5	$[M+H]^+$	-3.55	252.0998	O=C(OC)/C=C/C1=CC(OC)=C(C(OC)=C1)OC
1-(1-oxo-8-hydroxynonadienyl)piperidine	9.49	238.1817	C14H23NO2	$[M+H]^+$	6.33	237.1729	CC(O)CC\C=C\C=C\C(=O)N1CCCCC1
N-isobutyloctadienamide	9.39	218.1526	C12H21NO	$[M+Na]^+$	5.05	195.1623	CCC\C=C\C=C\C(=O)NCC(C)C
6-hydroxypellitorine	12.22	222.1848	C14H25NO2	$[M+H-H_2O]^+$	-1.87	239.1885	CCCCC(O)\C=C\C=C\C(=O)NCC(C)C
N-isobutyloctadeca-2,4,12-trienamide	19.07	356.2906	C22H39NO	$[M+Na]^+$	-4.97	333.3032	CCCCC\C=C/CCCCCC\C=C\C=C\C(=O)NCC(C)C
N-isobutyloctadeca-2,4,12-trienamide (isomer)	19.77	334.3086	C22H39NO	$[M+H]^+$	-5.52	333.3032	CCCCC\C=C/CCCCCC\C=C\C=C\C(=O)NCC(C)C
1-(eicosa-2E,4E,12Z-trienoyl)piperidine	20.81	374.3402	C25H43NO	$[M+H]^+$	-4.25	373.3345	CCCCC\C=C/CCCCCCCC\C=C\C=C\C(=O)N1CCCCC1
1-(eicosa-2E,14Z-dienoyl)piperidine	20.56	376.3561	C25H45NO	$[M+H]^+$	-3.57	375.3501	CCCCC\C=C/CCCCCCCCCC\C=C\C(=O)N1CCCCC1
pellitorine	12.77	224.1978	C14H25NO	$[M+H]^+$	-13.81	223.1936	CCCCC\C=C\C=C\C(=O)NCC(C)C
1-(piperidin-1-yl)dodeca-2,4-dien-1-one	15.84	264.2320	C17H29NO	$[M+H]^+$	-0.89	263.2249	CCCCCCC\C=C\C=C\C(=O)N1CCCCC1
N-2'-Methylbutyl-2,4-decadienamide	13.20	238.2175	C15H27NO	$[M+H]^+$	4.19	237.2093	CCCCC\C=C\C=C\C(=O)NCC(C)CC

3,4-methylenedioxycinnamaldehyde	16.02	177.0552	C10H8O3	$[M+H]^+$	3.38	176.0473	O=CC=CC1=CC=C2OCOC2=C1
N-isobutyldodeca-2,4-dienamide	15.00	252.2306	C16H29NO	$[M+H]^+$	-6.29	251.2249	CCCCCCC\C=C\C=C\C(=O)NCC(C)C
N-tetradecadienoyl]-piperidine	18.08	292.2608	C19H33NO	$[M+H]^+$	-9.17	291.2562	CCCCCCCCC\C=C\C=C\C(=O)N1CCCCC1
N-Isobutyl-2,4,8-eicosatrienamide	20.31	362.3401	C24H43NO	$[M+H]^+$	-4.43	361.3345	CCCCCCCCCCC\C=C/CC\C=C\C=C\C(=O)NCC(C)C
N-isobutylhexadeca-2,4-dienamide	18.87	308.2918	C20H37NO	$[M+H]^+$	-9.68	307.2875	CCCCCCCCCCC\C=C\C=C\C(=O)NCC(C)C
piperlongumine B	15.45	344.2931	C21H39NO	$[M+Na]^+$	2.17	321.3032	CCCCCCCCCCCCC\C=C\C(=O)N1CCCCC1
piperlongimin A	19.17	332.2942	C20H39NO	$[M+Na]^+$	5.33	309.3032	CCCCCCCCCCCCC\C=C\C(=O)NCC(C)C
1-(octadeca-2,4-dienoyl)piperidine	19.57	348.3255	C23H41NO	$[M+H]^+$	-1.6	347.3188	CCCCCCCCCCCCC\C=C\C=C\C(=O)N1CCCCC1
N-isobutyloctadeca-2,4-dienamide	19.57	336.3245	C22H41NO	$[M+H]^+$	-4.73	335.3188	CCCCCCCCCCCCC\C=C\C=C\C(=O)NCC(C)C
N-isobutyl-eicosa-2,4-dienamide	20.56	364.3552	C24H45NO	$[M+H]^+$	-5.94	363.3501	CCCCCCCCCCCCCCC\C=C\C=C\C(=O)NCC(C)C
aphanamol II	20.71	219.1749	C15H24O2	$[M+H-H_2O]^+$	2.73	236.1776	[H][C@@]12C=C(CC[C@@H](O)[C@]1(C)CC[C@H]2C(C)C)C=O
N-5-(4-hydroxy-3-methoxyphenyl)-2E-pentenoyl piperidine	10.88	290.1730	C17H23NO3	$[M+H]^+$	-7.01	289.1678	COC1=C(O)C=CC(CC\C=C\C(=O)N2CCCCC2)=C1
piplartine	9.29	318.1320	C17H19NO5	$[M+H]^+$	-4.91	317.1263	COC1=CC(\C=C\C(=O)N2CCC=CC2=O)=CC(OC)=C1OC
1-(1-Oxo-8-hydroxy-2,4-nonadienyl)piperidine	8.88	238.1810	C14H23NO2	$[M+H]^+$	3.36	237.1729	COC1=CC(\C=C\C(=O)N2CCCCC2)=CC(OC)=C1OC
3-(3,4,5-trimethoxyphenyl)acrylic acid	6.10	239.0920	C12H14O5	$[M+H]^+$	2.50	238.0841	OC(/C=C/C1=CC(OC)=C(C(OC)=C1)OC)=O
3β,4α-dihydroxy-2-piperidinone	5.07	132.0666	C5H9NO3	$[M+H]^+$	8.14	131.0582	O[C@H]1CCNC(=O)[C@@H]1O
piperlongramide C	10.73	593.2609	C34H38N2O6	$[M+Na]^+$	-2.14	570.273	O=C([C@H]1C=CC(C(\C=C/C2=CC3=C(OCO3)C=C2)[C@@H]1C(=O)N1CCCCC1)C1=CC=C2OCOC2=C1)N1CCCCC1
Chabamide	12.27	571.2765	C34H38N2O6	$[M+H]^+$	-6.62	570.273	O=C([C@H]1C=CC(C(\C=C/C2=CC3=C(OCO3)C=C2)[C@@H]1C(=O)N1CCCCC1)C1=CC=C2OCOC2=C1)N1CCCCC1
Pipernigramide E	12.67	571.2768	C34H38N2O6	$[M+H]^+$	-6.12	570.273	O=C([C@H]1C=CC(C(\C=C/C2=CC3=C(OCO3)C=C2)[C@@H]1C(=O)N1CCCCC1)C1=CC=C2OCOC2=C1)N1CCCCC1
Dipiperamide F	12.97	571.2757	C34H38N2O6	$[M+H]^+$	-8.08	570.273	O=C([C@H]1C=CC(C(\C=C/C2=CC3=C(OCO3)C=C2)[C@@H]1C(=O)N1CCCCC1)C1=CC=C2OCOC2=C1)N1CCCCC1
Nigramide B	13.41	571.2762	C34H38N2O6	$[M+H]^+$	-7.14	570.273	O=C([C@H]1C=CC(C(\C=C/C2=CC3=C(OCO3)C=C2)[C@@H]1C(=O)N1CCCCC1)C1=CC=C2OCOC2=C1)N1CCCCC1
Piperettine	12.07	312.1582	C19H21NO3	$[M+H]^+$	-3.84	311.1521	O=C(\C=C\C=C\C=C\C1=CC=C2OCOC2=C1)N1CCCCC1
Piperyline	11.92	272.1283	C16H17NO3	$[M+H]^+$	0.73	271.3160	O=C(N1CCCC1)/C=C/C=C/C(C=C2)=CC3=C2OCO3
Dehydropipernonaline	10.13	340.1895	C21H25NO3	$[M+H]^+$	-3.46	339.1834	O=C(\C=C\C=C\CC\C=C\C1=CC=C2OCOC2=C1)N1CCCCC1
Dehydropipernonaline isomer	13.26	340.1867	C21H25NO3	$[M+H]^+$	-11.85	339.1834	O=C(\C=C\C=C\CC\C=C\C1=CC=C2OCOC2=C1)N1CCCCC1
Piperamide-C9:3	12.07	326.1739	C20H23NO3	$[M+H]^+$	-3.67	325.1678	O=C(\C=C\C=C\CC\C=C\C1=CC2=C(OCO2)C=C1)N1CCCC1
piperundecalidine	15.05	368.2185	C23H29NO3	$[M+H]^+$	-9.53	367.2147	O=C(\C=C\C=C\CCCC\C=C\C1=CC2=C(OCO2)C=C1)N1CCCCC1
piperchabamide C	16.79	396.2510	C25H33NO3	$[M+H]^+$	-5.97	395.246	O=C(\C=C\C=C\CCCCCC\C=C\C1=CC2=C(OCO2)C=C1)N1CCCCC1

piperlongumamide D	18.38	424.2822	C27H37NO3	[M+H]$^+$	-5.68	423.2773	O=C(\C=C\C=C\CCCCCCCC\C=C\C1=CC2=C(OCO2)C=C1)N1CCCCC1
4,5-dihydropiperine	10.28	310.1397	C17H21NO3	[M+Na]$^+$	-5.34	287.1521	O=C(\C=C\CCC1=CC=C2OCOC2=C1)N1CCCCC1
Pipernolanine	13.71	342.2017	C21H27NO3	[M+H]$^+$	-13.63	341.1991	O=C(\C=C\CCCC\C=C\C1=CC2=C(OCO2)C=C1)N1CCCCC1
piperchabamide B	15.60	392.2162	C23H31NO3	[M+Na]$^+$	-8.79	369.2304	O=C(\C=C\CCCCCC\C=C\C1=CC2=C(OCO2)C=C1)N1CCCCC1
piperchabamide C isomer	17.33	398.2667	C25H35NO3	[M+H]$^+$	-5.84	397.2617	O=C(\C=C\CCCCCCCC\C=C\C1=CC2=C(OCO2)C=C1)N1CCCCC1
voleneol	17.69	221.1902	C15H26O2	[M+H-H_2O]$^+$	0.90	238.1933	[H][C@@]12[C@@H](O)[C@@H](CC[C@@]1(C)[C@H](O)CCC2=C)C(C)C
Piperine isomer	10.73	308.1255	C17H19NO3	[M+Na]$^+$	-0.57	285.1365	O=C(C=CC=CC1=CC2=C(OCO2)C=C1)N1CCCCC1
Asarinin	14.05	355.1157	C20H18O6	[M+H]$^+$	-5.35	354.1103	[H][C@@]12CO[C@H](C3=CC4=C(OCO4)C=C3)[C@]1([H])CO[C@H]2C1=CC2=C(OCO2)C=C1
episesamin	14.35	355.1168	C20H18O6	[M+H]$^+$	-2.25	354.1103	[H][C@]12CO[C@@H](C3=CC=C4OCOC4=C3)[C@@]1([H])CO[C@@H]2C1=CC2=C(OCO2)C=C1
pipercyclobutanamide H	16.19	627.3384	C38H46N2O6	[M+H]$^+$	-7.1	626.3356	O=C(C=CC1[C@H](CCCCC=CC2=CC3=C(OCO3)C=C2)C([C@@H]1C1=CC2=C(OCO2)C=C1)C(=O)N1CCCCC1)N1CCCCC1
methyl piperate	15.10	233.0797	C13H12O4	[M+H]$^+$	-4.71	232.0736	COC(=O)\C=C\C=C/C1=CC2=C(OCO2)C=C1
1-[(E)-7-(3,4-methylenedioxyphenyl)-6-heptenoyl]pyrrolidine	11.28	302.1725	C18H23NO3	[M+H]$^+$	-9.41	301.1678	O=C(CCCC\C=C\C1=CC2=C(OCO2)C=C1)N1CCCC1
Piperolein B	14.26	344.2205	C21H29NO3	[M+H]$^+$	-4.52	343.2147	O=C(CCCCCC\C=C\C1=CC=C2OCOC2=C1)N1CCCCC1
erythro-1-[1-oxo-9(3,4-methylenedioxyphenyl)-8,9-dihydroxy-2E-nonenyl]-piperidine	8.25	398.1951	C21H29NO5	[M+Na]$^+$	3.3	375.2046	OC(CCCC\C=C\C(=O)N1CCCCC1)C(O)C1=CC=C2OCOC2=C1
trans-cinnamyl β-D-glucopyranoside	12.67	593.2584	C15H20O6	[2M+H]$^+$	-1.37	296.126	OC[C@H]1O[C@@H](OC\C=C\C2=CC=CC=C2)[C@H](O)[C@@H](O)[C@@H]1O

2. Spectra of all compounds

2.1 Compound **1** in MeOH-***d₄***

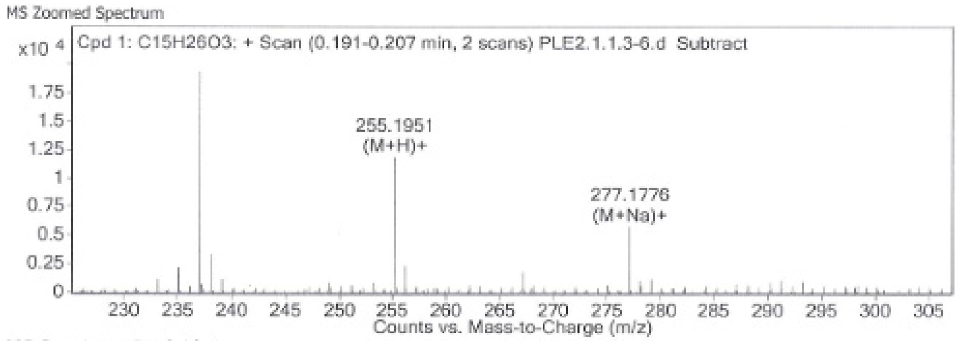

MS Spectrum Peak List

m/z	*Calc m/z*	Diff(ppm)	z	Abund	Formula	Ion
197.1503				1214		
201.1632				140794		
201.2573				3736		
201.2909				3353		
201.3306				1783		
202.1668				21243		
203.1737				3702		
255.1951	255.1955	-1.3	1	11904	C15 H27 O3	(M+H)+
256.1986	256.1989	-1.15	1	2321	C15 H27 O3	(M+H)+

Figure S2. (+)-HR-ESI-MS of compound **1**

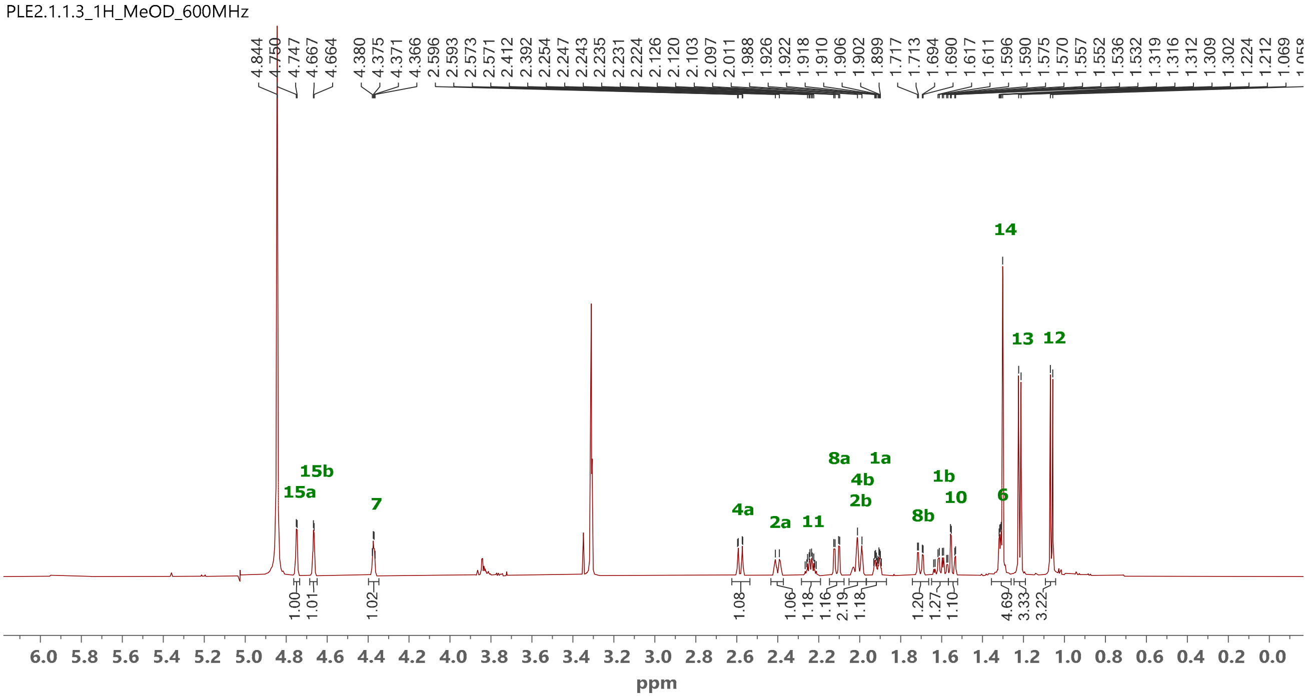

Figure S3. ^{1}H NMR spectrum of compound **1** in MeOH-d_4 (600 MHz)

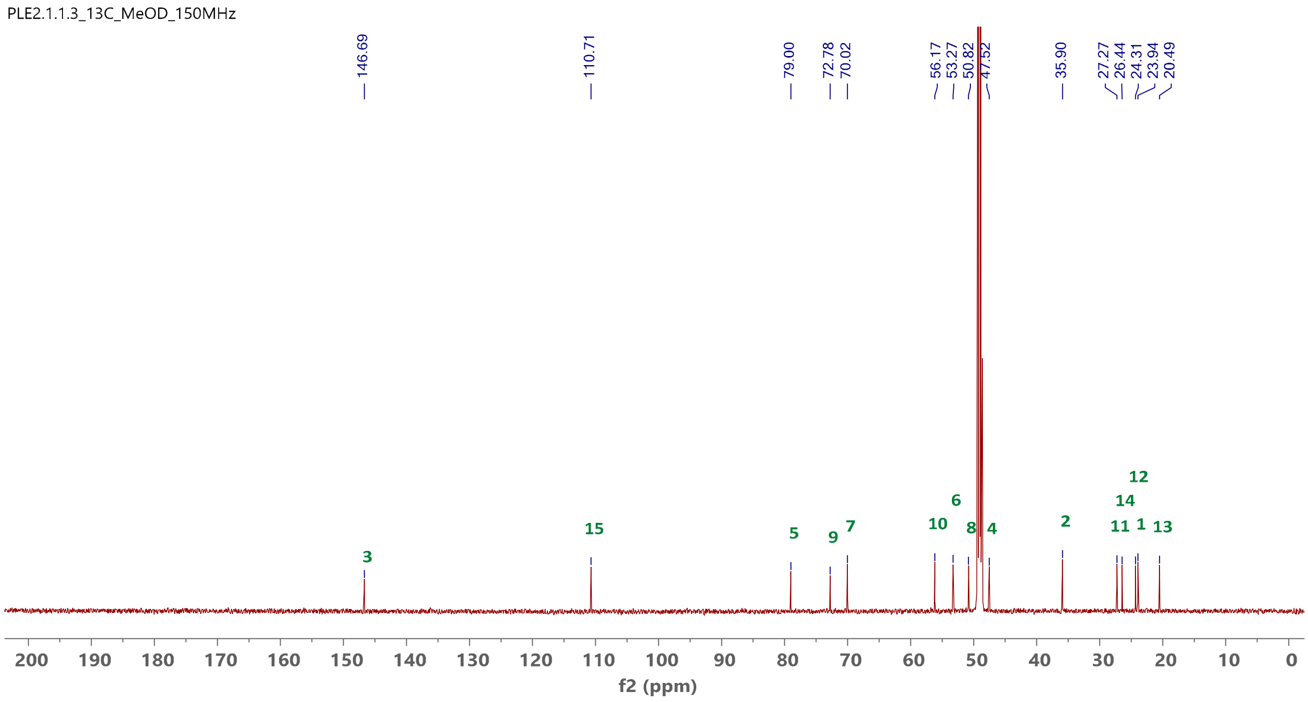

Figure S4. ^{13}C NMR spectrum of compound **1** in MeOH-d_4 (150 MHz)

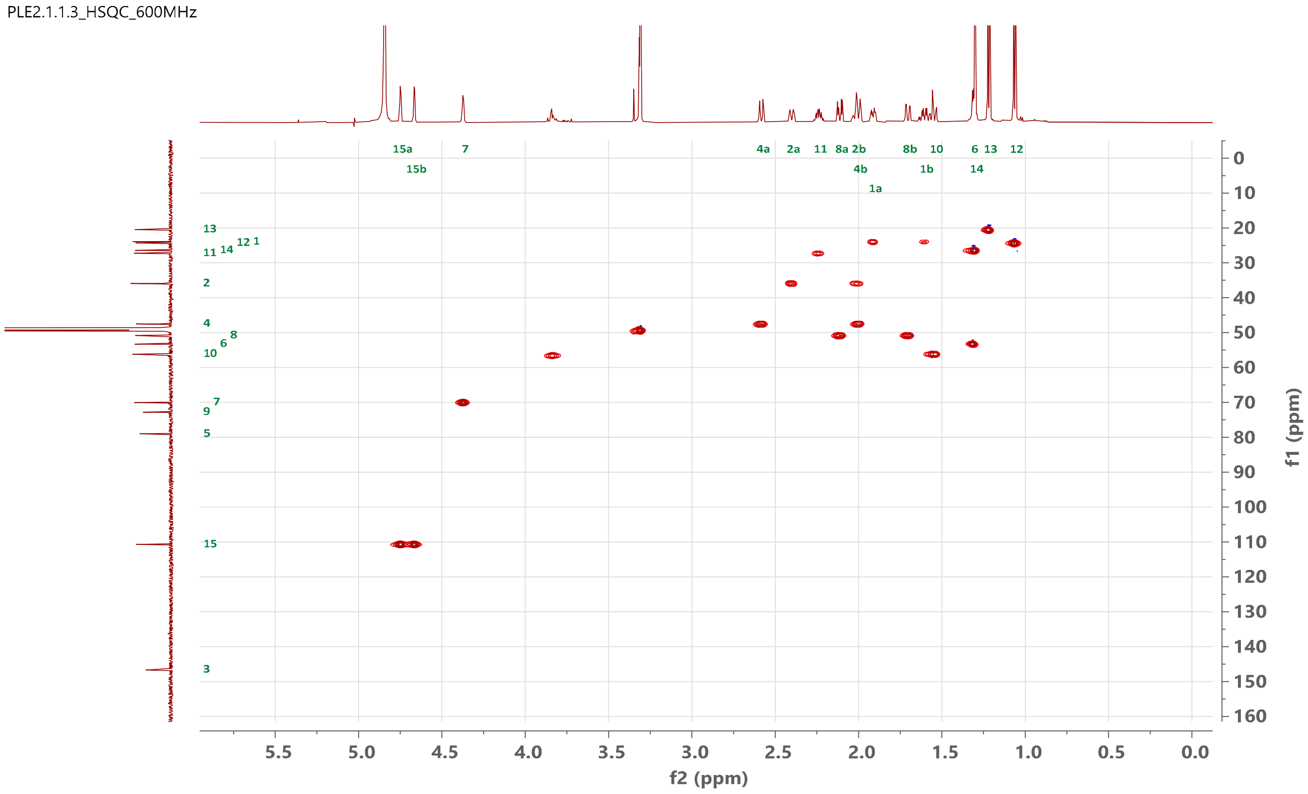

Figure S5. HSQC spectrum of compound **1** in MeOH-d_4 (600 MHz)

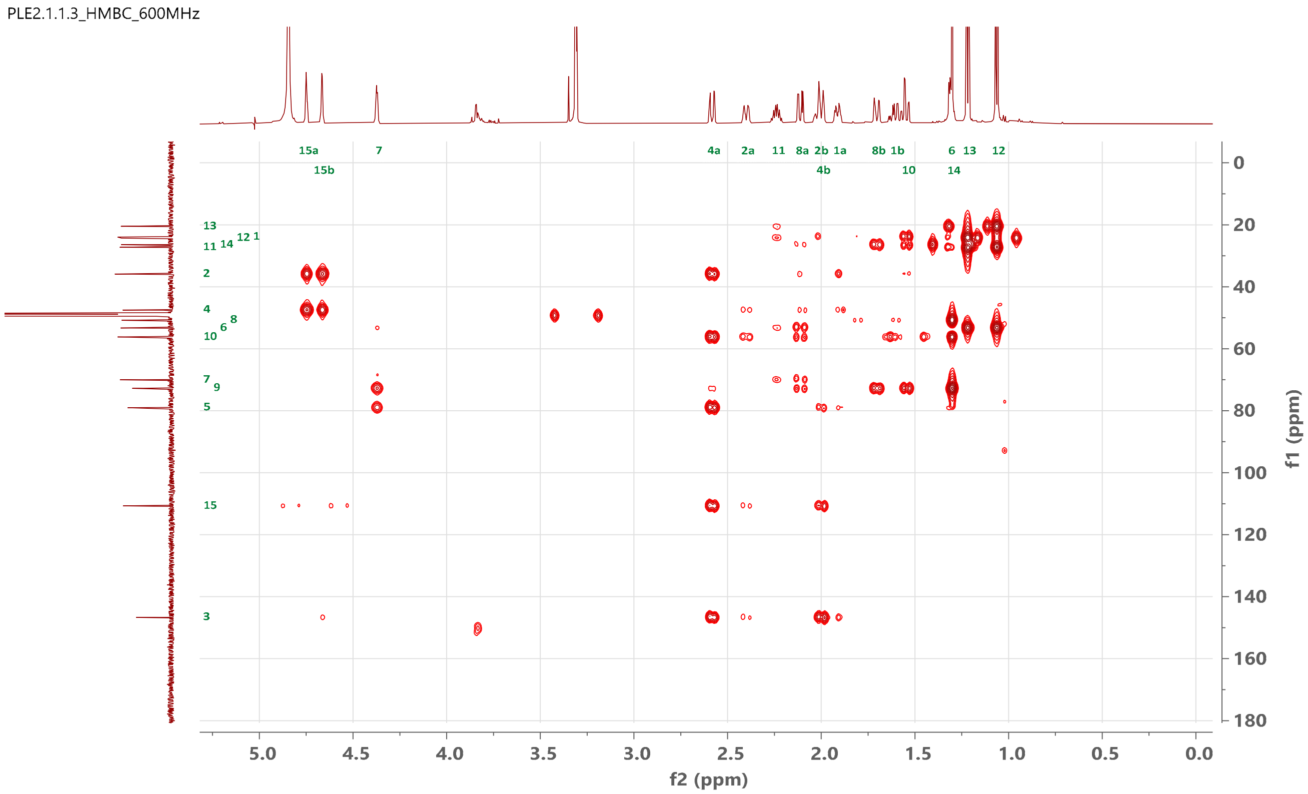

Figure S6. HMBC spectrum of compound **1** in MeOH-d_4 (600 MHz)

2.2 Compound **1** in DMSO-d_6

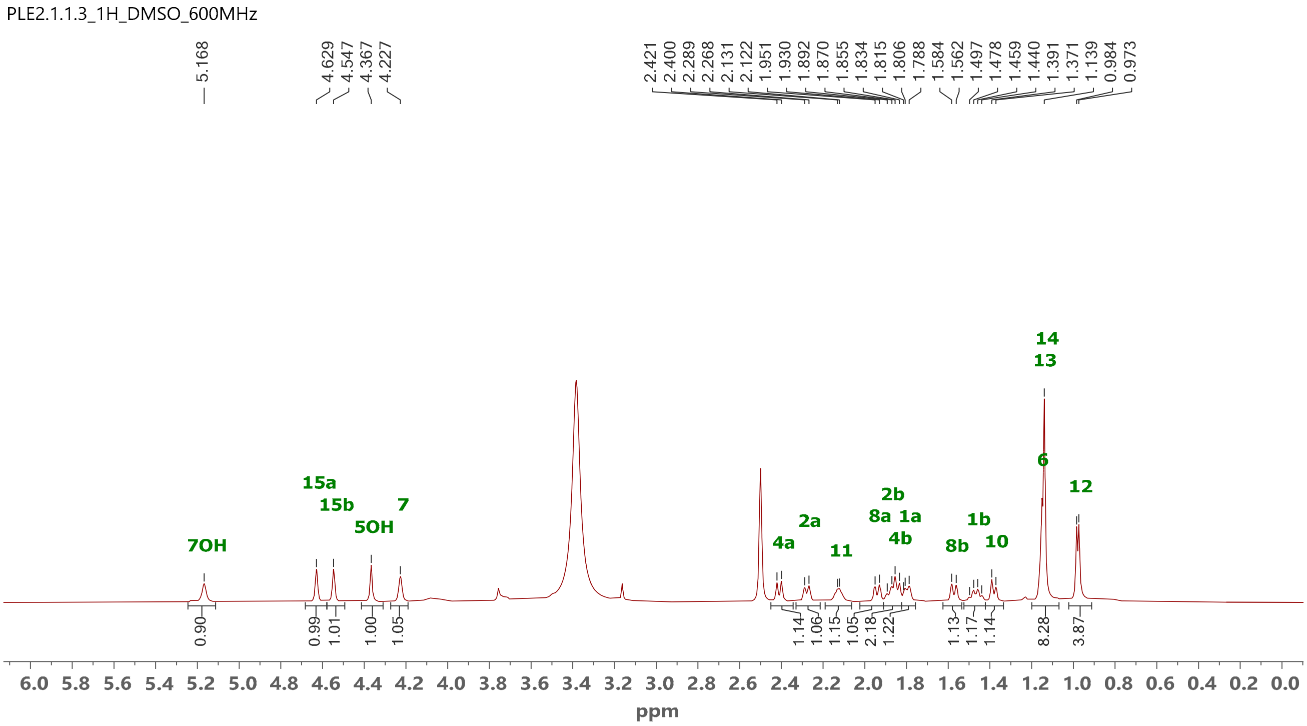

Figure S7. ^{1}H NMR spectrum of compound **1** in DMSO-d_6 (600 MHz)

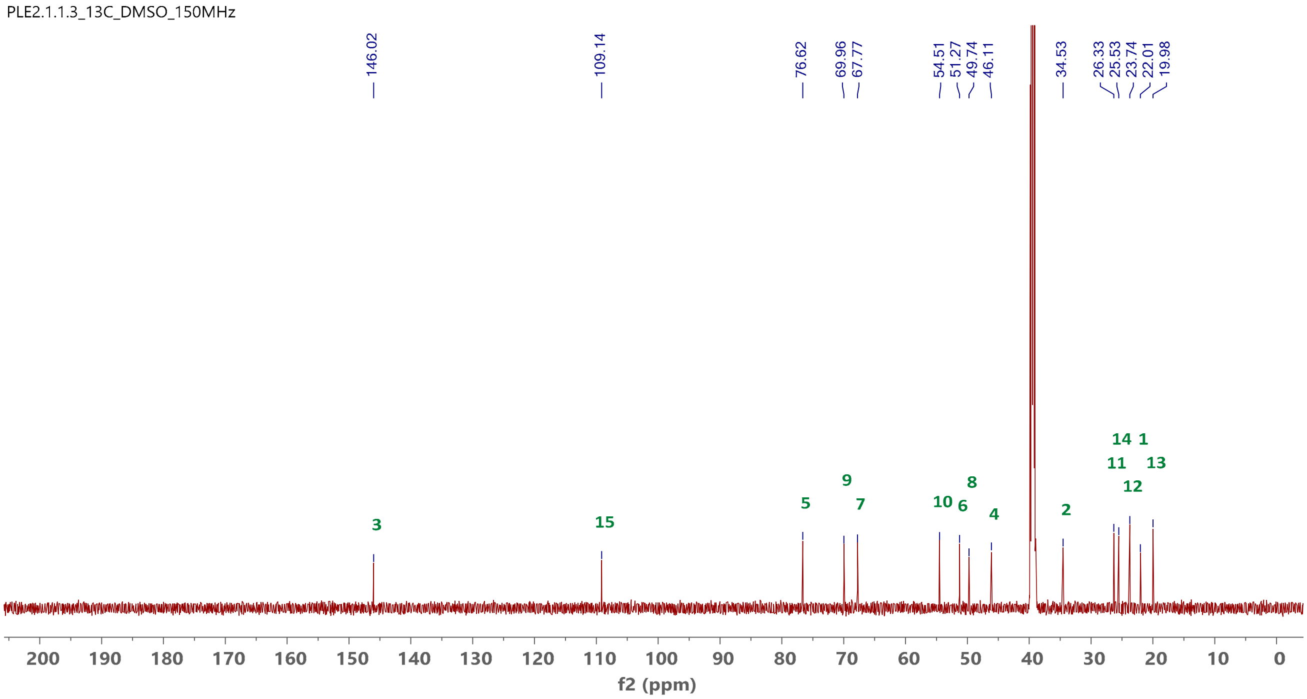

Figure S8. ^{13}C NMR spectrum of compound **1** in DMSO-d_6 (150 MHz)

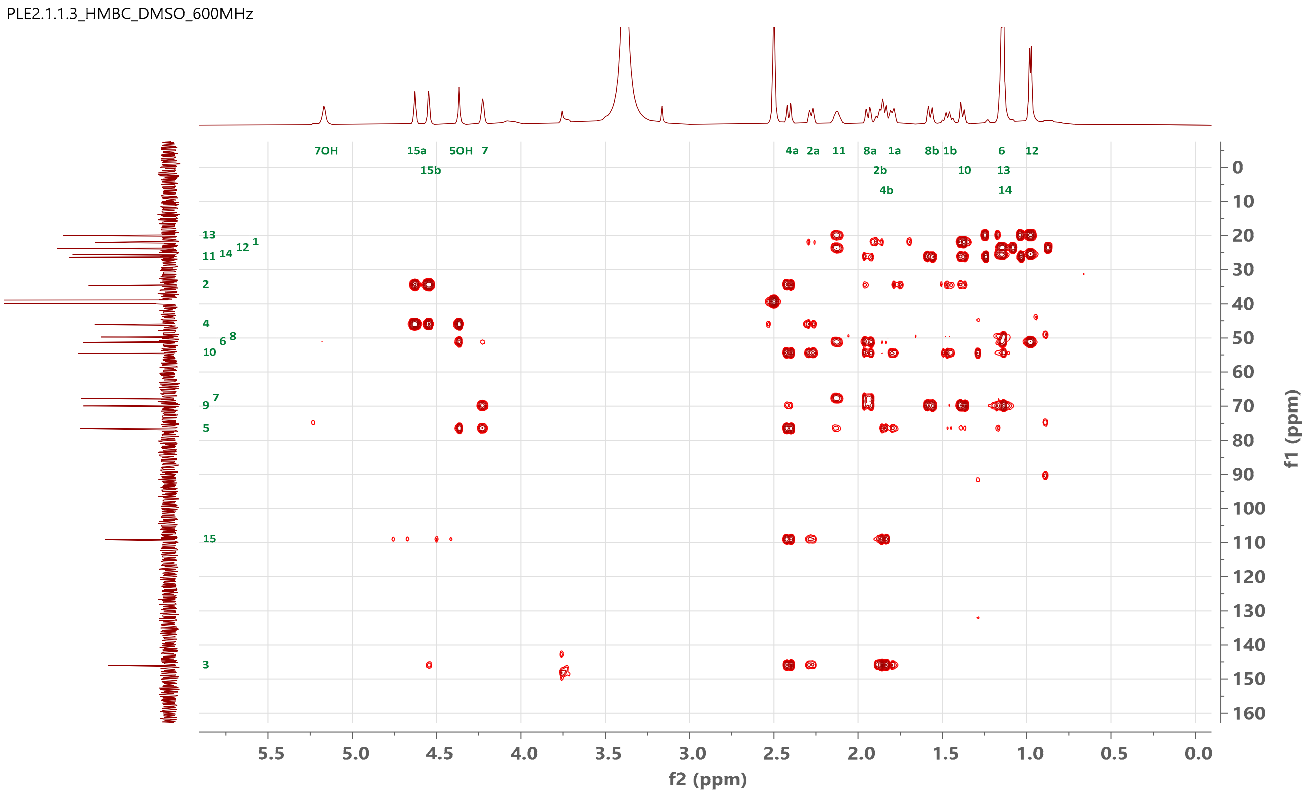

Figure S9. HMBC spectrum of compound **1** in DMSO-d_6 (600 MHz)

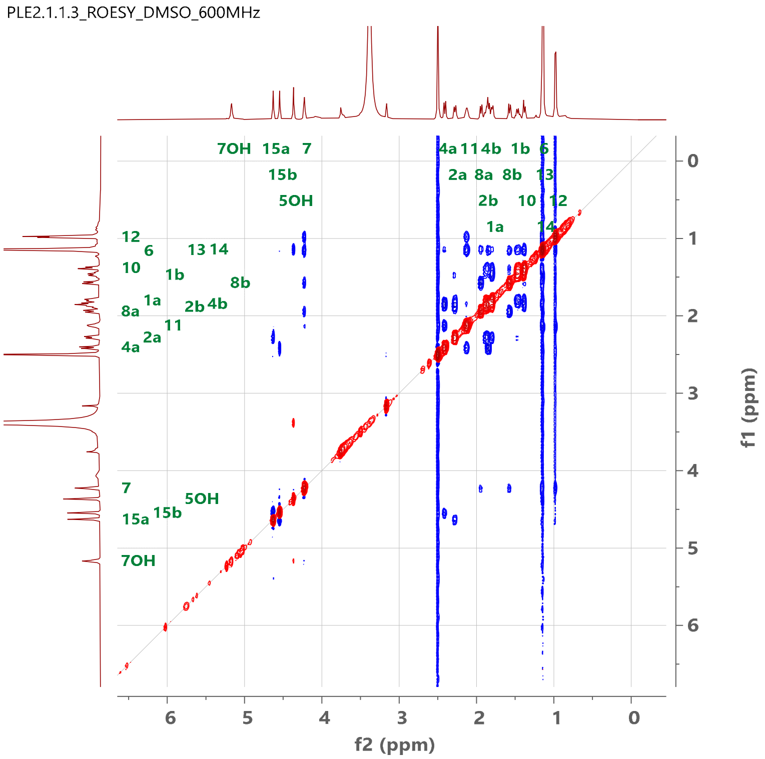

Figure S10. ROESY spectrum of compound **1** in DMSO-d_6 (600 MHz)

2.3 Compound **2** in MeOH-$\boldsymbol{d_4}$

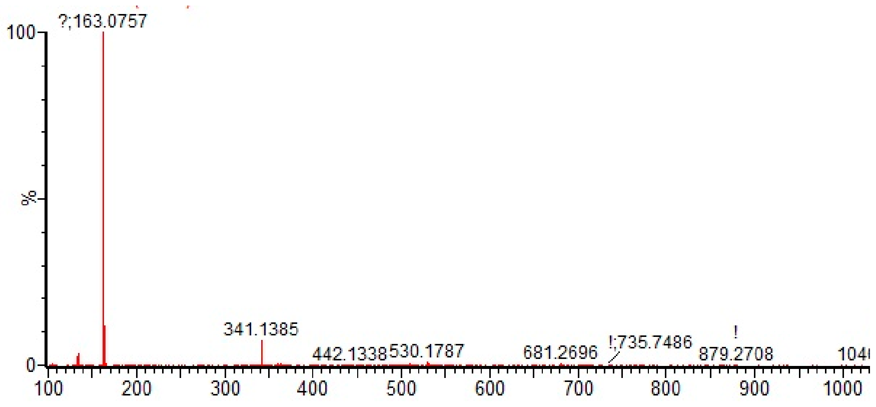

Figure S11. (+)-HR-ESI-MS of compound **2**

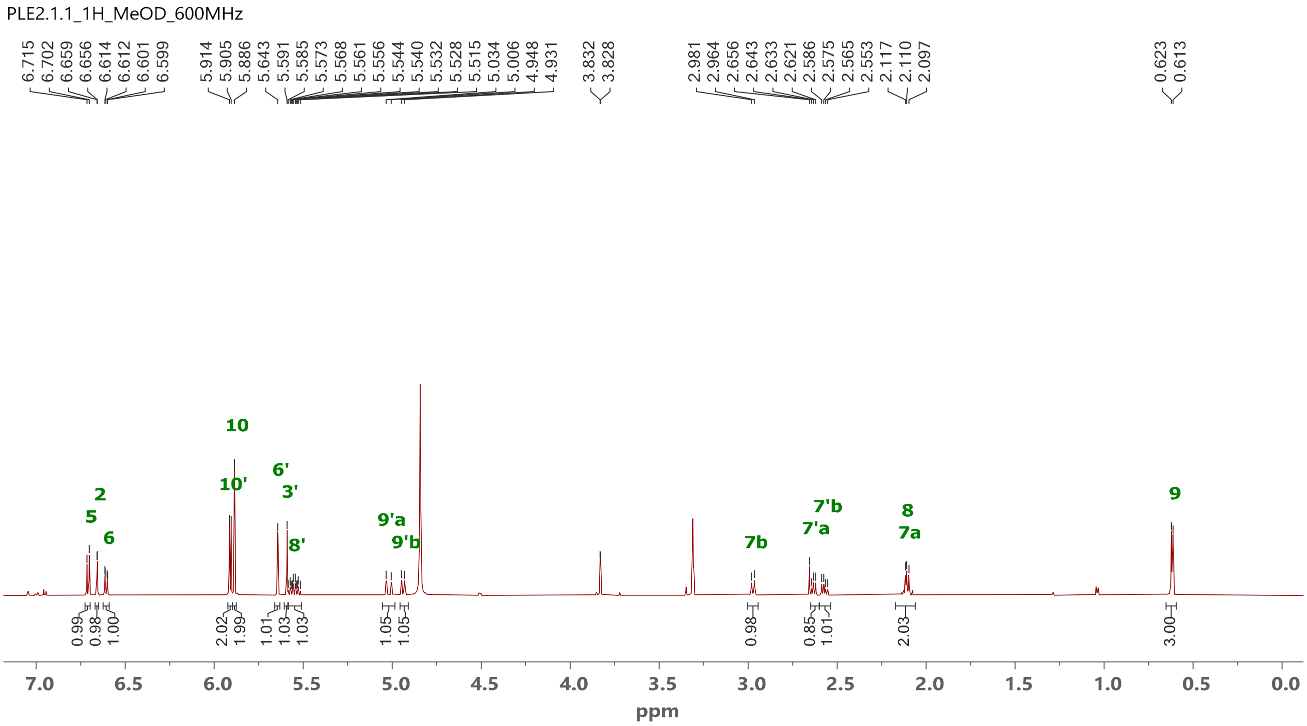

Figure S12. ^{1}H NMR spectrum of compound **2** in MeOH-d_4 (600 MHz)

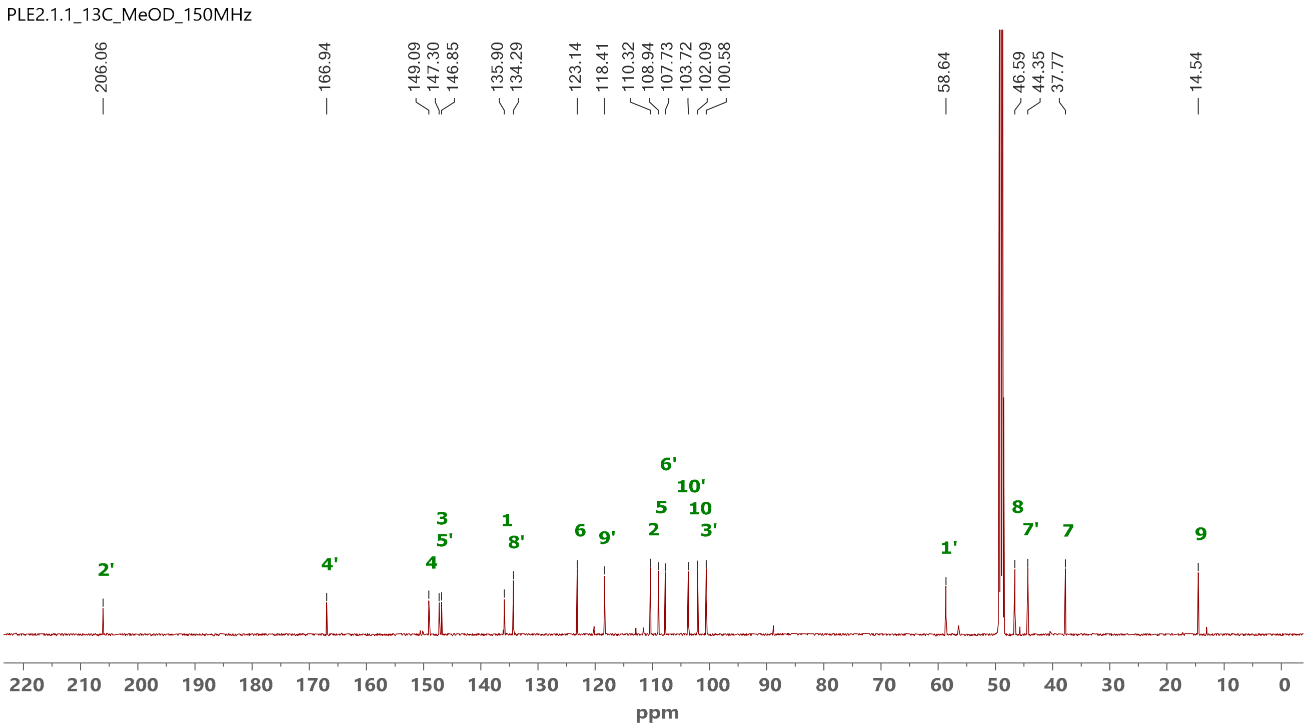

Figure S13. ^{13}C NMR spectrum of compound **2** in MeOH-d_4 (150 MHz)

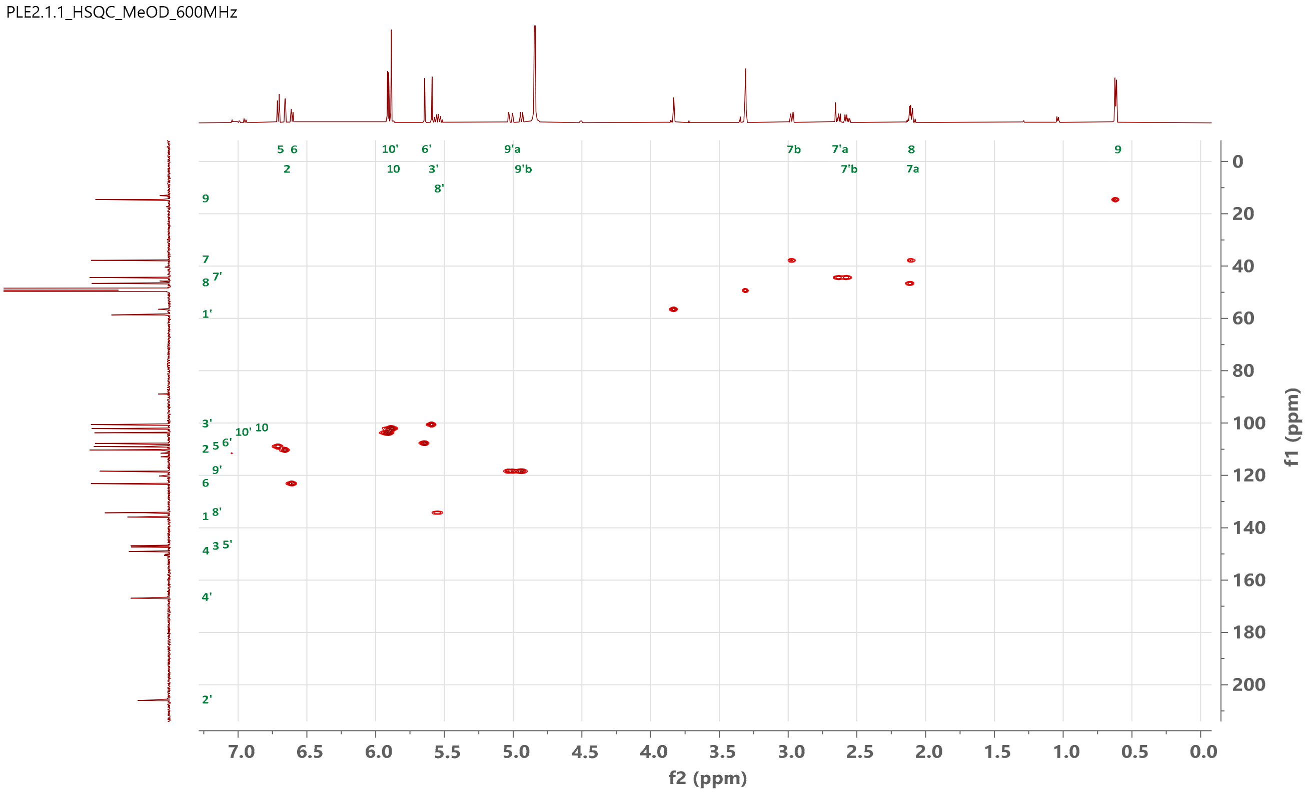

Figure S14. HSQC spectrum of compound **2** in MeOH-d_4 (600 MHz)

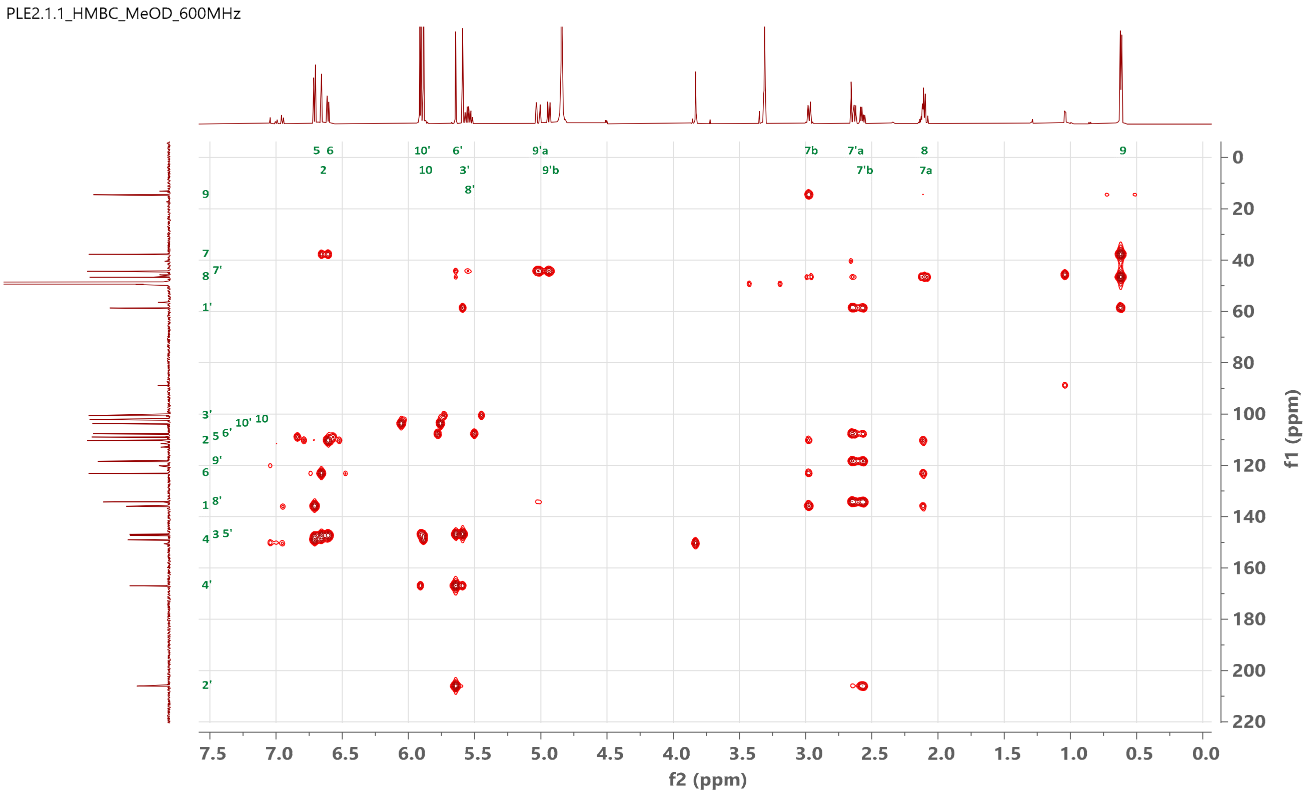

Figure S15. HMBC spectrum of compound **2** in MeOH-d_4 (600 MHz)

2.4 Compound **5** in MeOH-***d*₄**

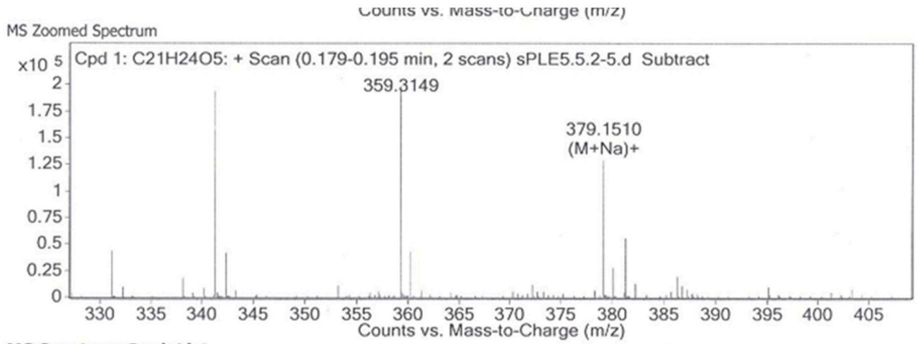

MS Spectrum Peak List

m/z	*Calc m/z*	Diff(ppm)	z	Abund	Formula	Ion
356.2854				3830		
357.1708	357.1697	3.09		5054	C21 H25 O5	(M+H)+
359.3149				197813		
359.4818				5031		
360.3179				42988		
361.3209				6342		
364.2733				4019		
379.151	379.1516	-1.45	1	130002	C21 H24 Na O5	(M+Na)+
380.1547	380.155	-0.88	1	28049	C21 H24 Na O5	(M+Na)+

Figure S16. (+)-HR-ESI-MS of compound **5**

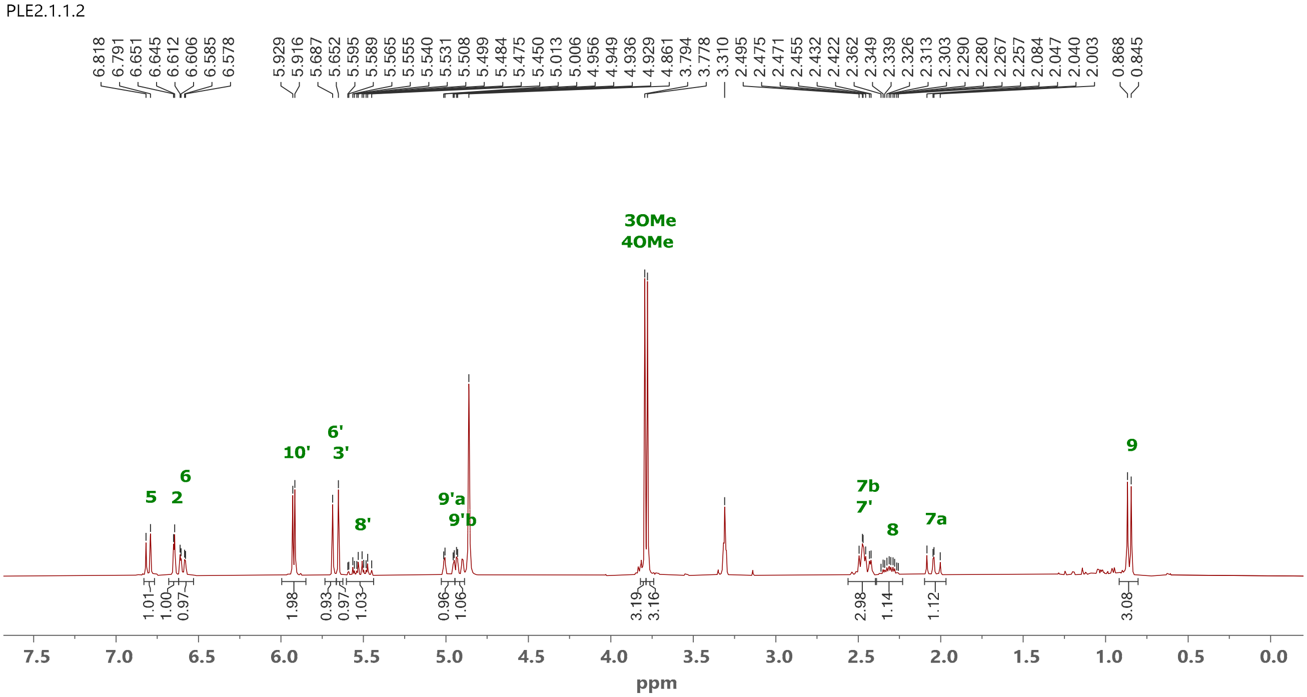

Figure S17. ¹H NMR spectrum of compound **5** in MeOH-*d*₄ (600 MHz)

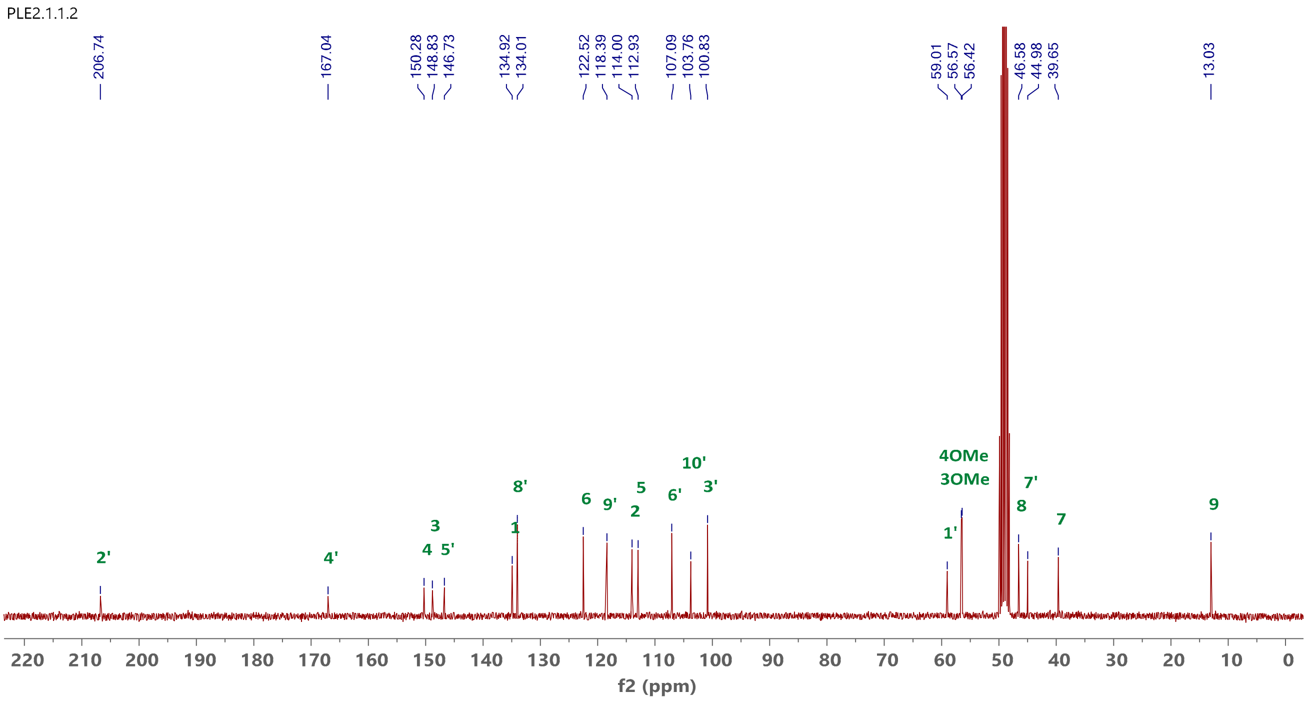

Figure S18. ^{13}C NMR spectrum of compound **5** in MeOH-d_4 (150 MHz)

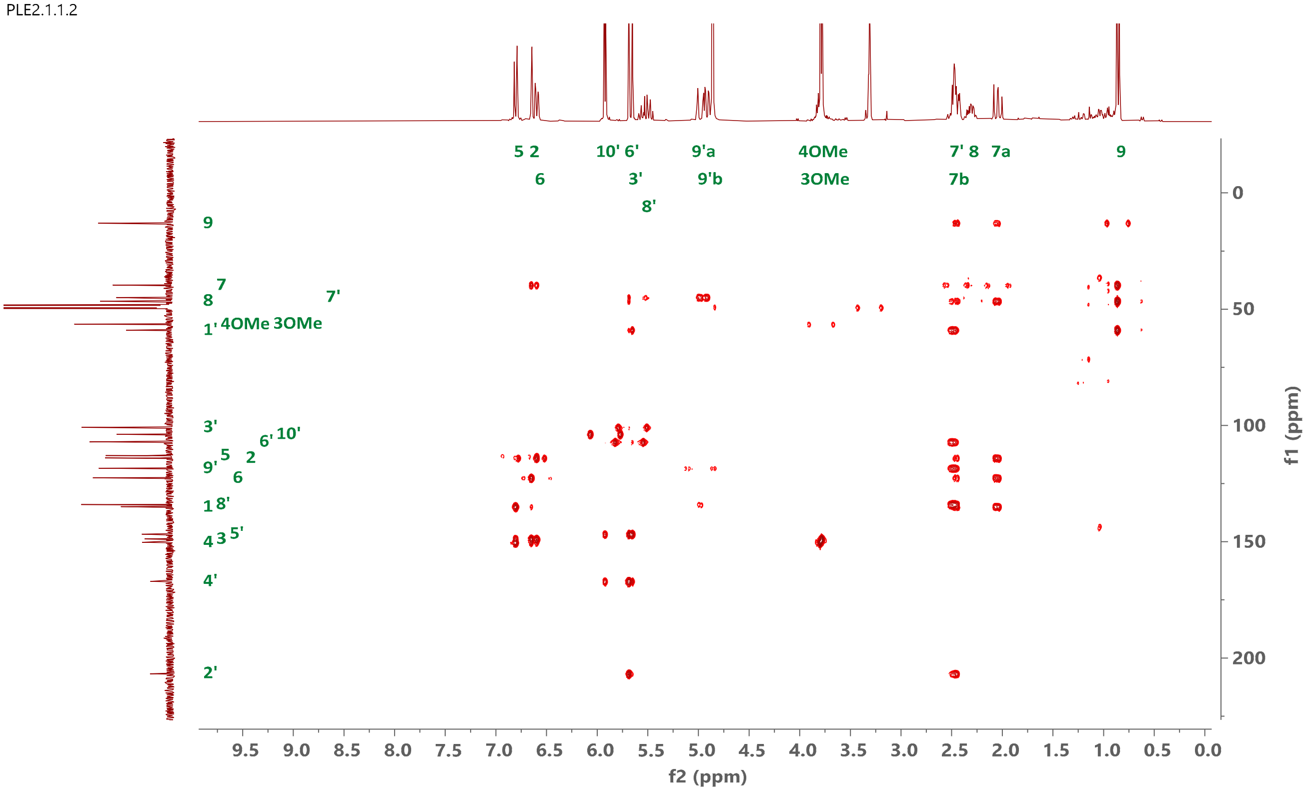

Figure S19. HMBC spectrum of compound **5** in MeOH-d_4 (600 MHz)

3. ECD Calculations

Table S2. Relative Gibbs free energy (ΔG) and Boltzmann population of 8*S*,1′*R*-**2**

**Conformer no.**	**ΔG (kcal/mol)**	**Boltzmann population (%)**
Conformer 11	0.000	40.33
Conformer 07	0.201	28.73
Conformer 05	0.744	11.50
Conformer 02	0.975	7.78
Conformer 15	1.347	4.15
Conformer 16	1.567	2.86
Conformer 13	1.630	2.57
Conformer 14	1.759	2.07

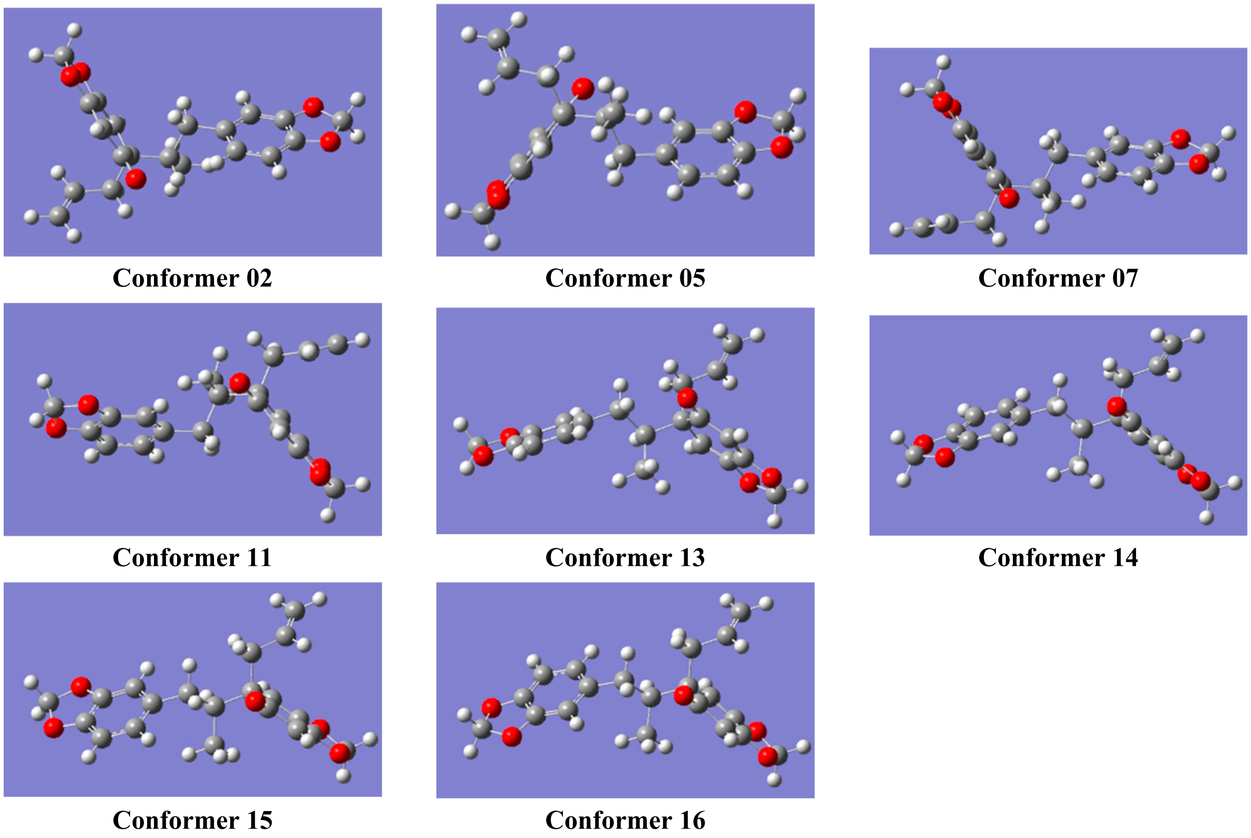

Figure S20. Energy-minimized conformers of 8*S*,1′*R*-**2** at the B3LYP/6-31G(d) in the gas phase

Table S3. Relative Gibbs free energy (ΔG) and Boltzmann population of 8*S*,1′*S*-**2**

**Conformer no.**	**ΔG (kcal/mol)**	**Boltzmann population (%)**
Conformer 20	0.000	28.09
Conformer 18	0.220	19.37
Conformer 13	0.366	15.15
Conformer 10	0.435	13.48
Conformer 14	0.783	7.50
Conformer 08	0.953	5.63
Conformer 12	0.998	5.21
Conformer 06	1.313	3.06
Conformer 09	1.431	2.51

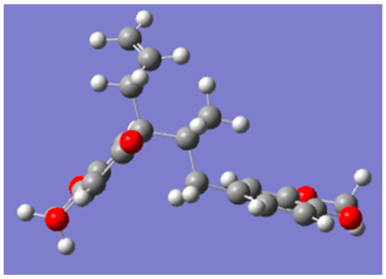

Conformer 06

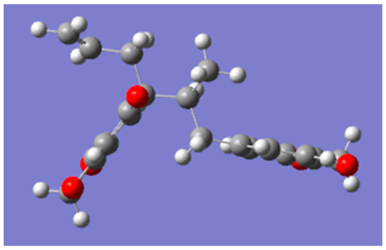

Conformer 08

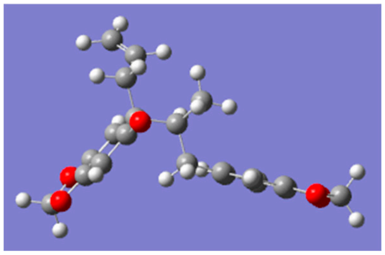

Conformer 09

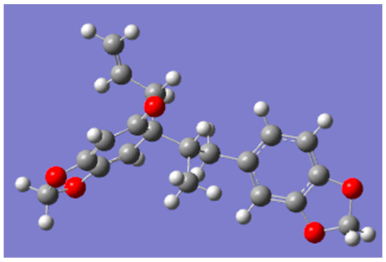

Conformer 10

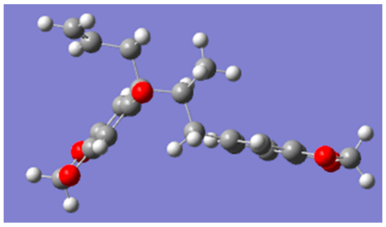

Conformer 12

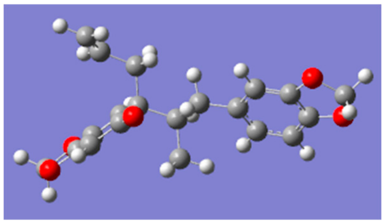

Conformer 13

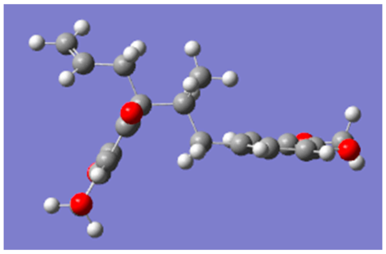

Conformer 14

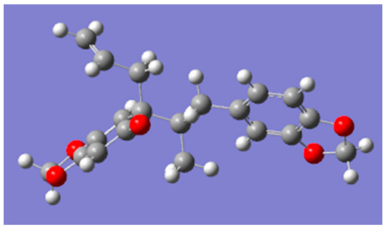

Conformer 18

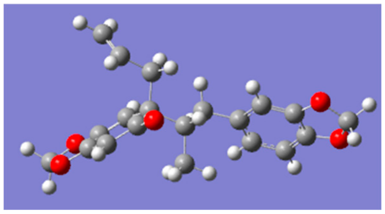

Conformer 20

Figure S21. Energy-minimized conformers of 8*S*,1′*S*-**2** at the B3LYP/6-31G(d) in the gas phase
